# Cortistatin protects against intervertebral disc degeneration through targeting mitochondrial ROS-dependent NLRP3 inflammasome activation

**DOI:** 10.7150/thno.45359

**Published:** 2020-05-27

**Authors:** Yunpeng Zhao, Cheng Qiu, Wenhan Wang, Jiangfan Peng, Xiang Cheng, Yangtao Shangguan, Mingyang Xu, Jiayi Li, Ruize Qu, Xiaomin Chen, Suyi Jia, Dan Luo, Long Liu, Peng Li, Fengjin Guo, Krasimir Vasilev, Liang Liu, John Hayball, Shuli Dong, Xin Pan, Yuhua Li, Linlin Guo, Lei Cheng, Weiwei Li

**Affiliations:** 1 Department of Orthopaedic Surgery, Qilu Hospital, Cheeloo College of Medicine, Shandong University, Jinan, Shandong, 250012, P. R. China.; 2 Cheeloo College of Medicine, Shandong University, Jinan, Shandong, 250012, P. R. China.; 3 College of Stomatology, Qingdao University, Qingdao, Shandong, 266071, P. R. China.; 4 Department of Pathology, Qilu Hospital, Cheeloo College of Medicine, Shandong University, Jinan, Shandong, 250012, P. R. China.; 5 Department of Cell Biology and Genetics, Core Facility of Development Biology, Chongqing Medical University, Chongqing, 400016, P. R. China.; 6 Future Industries Institute, University of South Australia, Mawson Lakes Campus, Mawson Lakes 5095, Australia.; 7 School of Engineering, University of South Australia, Mawson Lakes Campus, Mawson Lakes 5095, Australia.; 8 Experimental Therapeutics Laboratory, University of South Australia Cancer Research Institute, Adelaide SA 5000, Australia.; 9 Robinson Research Institute and Adelaide Medical School, University of Adelaide, Adelaide SA 5005, Australia.; 10 Key Laboratory of Colloid and Interface Chemistry (Shandong University), Ministry of Education, Jinan, 250100, P. R. China.

**Keywords:** Intervertebral disc degeneration, cortistatin, mitochondrial ROS, NF-κB signalling pathway, NLRP3

## Abstract

**Background:** Intervertebral disc (IVD) degeneration is a common degenerative disease that can lead to collapse or herniation of the nucleus pulposus (NP) and result in radiculopathy in patients.

**Methods:** NP tissue and cells were isolated from patients and mice, and the expression profile of cortistatin (CST) was analysed. In addition, ageing of the NP was compared between 6-month-old WT and CST-knockout (CST^-/-^) mice. Furthermore, NP tissues and cells were cultured to validate the role of CST in TNF-α-induced IVD degeneration. Moreover, *in vitro* and *in vivo* experiments were performed to identify the potential role of CST in mitochondrial dysfunction, mitochondrial ROS generation and activation of the NLRP3 inflammasome during IVD degeneration. In addition, NF-κB signalling pathway activity was tested in NP tissues and cells from CST^-/-^ mice.

**Results:** The expression of CST in NP cells was diminished in the ageing- and TNF-α-induced IVD degeneration process. In addition, compared with WT mice, aged CST^-/-^ mice displayed accelerated metabolic imbalance and enhanced apoptosis, and these mice showed a disorganized NP tissue structure. Moreover, TNF-α-mediated catabolism and apoptosis were alleviated by exogenous CST treatment. Furthermore, CST inhibited mitochondrial dysfunction in NP cells through IVD degeneration and suppressed activation of the NLRP3 inflammasome. *In vitro* and* ex vivo* experiments indicated that increased NF-κB pathway activity might have been associated with the IVD degeneration observed in CST^-/-^ mice.

**Conclusion:** This study suggests the role of CST in mitochondrial ROS and activation of the NLRP3 inflammasome in IVD degeneration, which might shed light on therapeutic targets for IVD degeneration.

## Introduction

Intervertebral disc (IVD) degeneration is one of the most prevalent musculoskeletal disorders and can lead to chronic lower back pain 1. IVD degeneration is a complicated process, and its mechanism and therapeutic strategies remain to be elucidated 2. Dysfunction of NP cells and degradation of the extracellular matrix (ECM) are critical features of IVD degeneration 3. During IVD degeneration, mechanical loading may give rise to ECM injury, which contributes to an abnormal microenvironment in NP cells 4. Moreover, inflammatory cytokine secretion is enhanced through ageing and thus triggers disordered metabolism and apoptosis in NP cells 5. Currently, maintenance of NP cell homeostasis and attenuation of ECM degradation have become potential therapeutic strategies for IVD degeneration, which is being widely investigated 6.

The ageing of NP cells is associated with dysfunction in various organelles, especially mitochondria 7. As the predominant part of the respiratory chain, mitochondria play a critical role in the homeostasis of various cell types 8. IVD degeneration, mechanical injury, cytokine induction and other stimuli can cause damage to the mitochondria in NP cells 9. Mitochondrial dysfunction induces the production of reactive oxygen species (ROS), which in turn leads to exaggerated inflammation, disordered metabolism and enhanced apoptosis in cells 10. Among the molecules triggered by mitochondrial ROS, the NLRP3 inflammasome has been extensively studied for its detrimental role in IVD degeneration 10,11. Activation of the NLRP3 inflammasome can elevate the production of IL-1β, which facilitates the secretion of metalloproteinases and causes degradation of the NP tissue 12,13. In addition, NLRP3 is linked to the mitochondrial apoptosis pathway, programmed cell death, and apoptosis through several mechanisms in NP cells 14.

Hitherto, accumulating evidence regarding IVD degeneration has pointed towards the involvement of NP cell changes driven by inflammatory cytokines 15. Various inflammatory cytokines have been reported to be released into the local degenerative milieu of the NP tissue 16. During this process, TNF-α generates an inflammatory microenvironment that is involved in dysfunction and the altered apoptosis of NP cells 17. TNF-α enhances the expression levels of several members of the MMP and ADAMTS families, which plays a detrimental role in the homeostasis of the disc matrix 18. The NF-κB signalling pathway is a critical pathway that facilitates the function of TNF-α, which has been extensively studied in IVD degeneration 19,20. Activation of the NF-κB signalling pathway promotes catabolism through the production of metalloproteases and enhances apoptosis through the release of apoptotic biomarkers 21. Moreover, NF-κB signalling pathway activation is associated with mitochondrial dysfunction, which exaggerates mitochondrial ROS and NLRP3 inflammasome activation 22-24.

Cortistatin (CST) is a cyclic neuropeptide that exhibits distinct properties in various physiological and disease processes 25,26. Relevant findings in several models of autoimmune diseases, including inflammatory bowel disease and rheumatoid arthritis, have revealed that CST may represent a natural, endogenous anti-inflammatory factor 27,28. Furthermore, CST is an attractive candidate for the pharmacological management of degenerative and inflammatory conditions 27,29,30. Moreover, a recent study indicated that CST improved metabolism, suppressed apoptosis and attenuated inflammation in TNF-α-induced chondrocytes, and CST was shown to fight against the degeneration of articular cartilage in osteoarthritis 31.

However, whether CST is involved in IVD degeneration is unknown. The objectives of this research were to elucidate the potential function and underlying mechanisms of CST in IVD degeneration.

## Materials and Methods

### Animals

All the animal experiments utilized in this study were cared for in accordance with the Institutional Animal Care and Use Committee of Shandong University (Shandong, China). Genetically identified cortistatin-knockout (CST^-/-^) mice with a C57BL/6 background were established by the National Resource Center of Model Mice (Nanjing, China). The murine *Cort* gene encodes the CST protein. To create CST-knockout mice by CRISPR/Cas9-mediated genome engineering, gRNA directed Cas9 endonuclease cleavage of the *Cort* gene to create double-strand breaks. These breaks were repaired by non-homologous end jointing (NHEJ), resulting in the deletion of *Cort* 31. Wild-type (WT) mice were purchased from the Experimental Animal Center of Shandong University. WT and CST^-/-^ mice at 2, 6, and 10 months of age were used for the experiments. Three-month-old Sprague-Dawley (SD) rats were purchased from the Animal Center of Shandong University. All of the animals were housed under controlled identical specific pathogen-free (SPF) standard environmental conditions (23 ± 2°C, 12 hours light/dark cycle) with free access to food and allowed to move freely.

### Human samples and ethics statement

Human NP specimens were obtained from 17 patients (8 males and 9 females; aged 24-74; mean age = 40.41 ± 15.9) with intervertebral disc degeneration (all with grade II or grade IV IVD degeneration) caused by different conditions, including lumbar spinal stenosis, spondylolisthesis and lumbar disc herniation, who underwent lumbar spine surgery (March 2015 to June 2018) at Qilu Hospital of Shandong University. The degree of IDD was assessed according to the modified Pfirrmann grading system 32. Information regarding the patients who supplied grade II (n = 10) and grade IV (n = 7) IVD samples is listed in **Table S1**. Human NP tissues and cells were isolated from the grade II group during surgery and stimulated with TNF-α or IL-1β for further experiments. This study was approved by the Medical Ethical Committee of Qilu Hospital of Shandong University. The patients involved in this study signed informed consent documents and voluntarily agreed to participate in this research.

### Culture of murine nucleus pulposus (NP) cells

In this study, both WT and CST^-/-^ mice were sacrificed by cervical vertebra dislocation and soaked in 75% ethyl alcohol for 10 min to sterilize the entire body. After the dorsal hair had been shaved entirely, the skin was dissected from the back, and the whole spine was separated. Intervertebral discs were isolated and submerged in culture medium with Hanks' buffer. The disc tissue was cut into pieces and placed in plastic culture dishes containing DMEM/F-12 medium (HyClone, Thermo Co., USA) supplemented with 10% foetal bovine serum (FBS, Gibco, USA), 1% 100 U/mL penicillin and 100 mg/mL streptomycin (HyClone, USA). All disc cultures were cultured in an incubator at 37°C under 5% CO_2_ and 95% air. The culture media were replaced when NP cell adherence was achieved. Later, the culture media were replaced every 2 days, and the cells were passaged when they reached 80-90% confluence 33.

### Culture of primary human nucleus pulposus cells

Human IVD samples were harvested from lumbar spine surgery patients in compliance with the requirements of Qilu Hospital, Shandong University. NP cells were isolated and cultured as previously reported 34. Briefly, after washing with sterile phosphate-buffered saline (PBS) 3 times, endplate cartilage and annulus fibrosus (AF) were meticulously eliminated from the human IVD tissues. IVD tissues were carefully cut into fragments approximately 1 mm^3^ in volume. Then, 0.25% trypsin (HyClone, Logan, USA) was used to digest the NP tissue for 30 minutes, followed by 0.2% type II collagen (Sigma-Aldrich, St. Louis, USA) for 4 hours. Then, NP cells were synchronously cultured (95% air, 5% CO2, 37°C) in DMEM/F-12 medium (HyClone, Thermo Co.) supplemented with 10% foetal bovine serum (FBS, Gibco, USA), 1% 100 U/mL penicillin and 100 mg/mL streptomycin (HyClone, USA) at pH 7.2. The culture medium was replaced every 3 days, and the cells were passaged when they reached 80-90% confluence. Second- or third-generation cells were used for the indicated experiments.

### Isolation and culture of complete mouse IVDs

This procedure was performed as previously reported with minor modifications 35,36. Mice were sacrificed by cervical dislocation, and the whole spine was extracted after verification of the lumbar spine location. The lumbar spines were completely immersed in sterile PBS containing 100 U/mL penicillin/streptomycin for 30 minutes. Then, the lumbar spines were washed with sterile PBS 3 times. Next, the lumbar spines were cut into pieces 5 mm in length while ensuring the integrity of the intervertebral disc. Finally, the disc specimens were carefully placed into 6-well plates and cultured with DMEM/F-12 culture medium (HyClone, Thermo Co., USA) containing 10% foetal bovine serum (FBS, Gibco, USA), 1% 100 U/mL penicillin and 100 mg/mL streptomycin (HyClone, USA). The indicated culture groups were stimulated with TNF-α at 24 h after isolation as previously reported 37.

### Isolation and culture of human IVD tissues

This procedure was conducted as previously reported and performed under sterile conditions 38,39. Human IVD tissues were derived from patients undergoing traumatic lumbar surgery. Briefly, the annulus fibrous and endplates were meticulously removed, and explants were cultured in DMEM/F-12 culture medium (HyClone, Thermo Co., USA) supplemented with 10% foetal bovine serum (FBS, Gibco, USA), 1% 100 U/mL penicillin and 100 mg/mL streptomycin (HyClone, USA). The indicated culture groups were stimulated with TNF-α at 24 h after isolation as previously reported 37.

### Rat model establishment

The rat IDD model was established as previously reported 14,40. The area between the eighth and ninth coccygeal vertebrae (Co8-Co9) in rats was punctured by using a 20-gauge needle. To eliminate the effect of injection, identical volume of PBS or CST (250 μg/kg body weight) was injected intraperitoneally every other day. All injections were implemented 3 days after the day of model establishment. Modelling was confirmed at four-week post-puncture by X-ray.

### Real-time PCR

The NP tissues were immediately snap-frozen in liquid nitrogen and stored at -80°C until mRNA extraction. Total RNA was separately extracted from the IVD tissues and NP cells of each indicated group. According to the manufacturer's recommended procedure, an RNAeasy^TM^ kit (R0026; Beyotime Biotechnology) was used to extract total RNA. Briefly, tissues were ground into a homogenate to which NP cells and lysis buffer were added. An identical volume of binding solution was then added, and the mixture was vortexed 3-5 times. Next, the mixture was purified over a column and centrifuged at 12000 ×g for 30 seconds, after which the supernatant was collected in a tube. Afterwards, washing buffer I was added to the supernatant, which was centrifuged at 12000 ×g for 30 seconds, after which the supernatant was discarded. Then washing buffer II was added to the pellet and centrifuged at 12000 ×g for 30 seconds, after which the supernatant was discarded. Washing buffer II was added again, and the process was repeated. Then, the sample was centrifuged at 15000 ×g for 2 minutes, and the residual liquid was removed. Finally, the extracted RNA was placed in an eluting tube to which 50 μL of eluent was added. The mixture was centrifuged at 16000 ×g for 30 seconds after 2-minute incubation at room temperature, and then the total RNA was extracted. Real-time PCR was carried out after the total RNA was extracted and reverse transcribed to cDNA using an RT-PCR kit (Vazyme Biotech, Nanjing, China) according to the manufacturer's instructions. PCR experiments were run in triplicate using 60 ng of cDNA per reaction and 10 μM forward and reverse primers with 2× ChamQ SYBR Color qPCR Master Mix (Vazyme Biotech) on a Bio-Rad CFX 96 system (Bio-Rad, California, USA). The nucleotide sequences of the primers are listed in **Tables S2 and S3**. The PCR cycling conditions consisted of an initial 30 seconds of denaturation at 95°C, followed by 40 cycles of 10 seconds of denaturation at 95°C and 30 seconds of annealing/elongation. The expression levels of target genes were normalized to GAPDH, and target gene expression was calculated by the 2^-ΔΔCT^ method. The control group was used for normalization, and the results are expressed as the fold change according to a previously published study 36.

### Western blot analysis

NP tissues isolated from WT and CST^-/-^ mice, as well as human NP tissues cultured *ex vivo*, were homogenized after the end of the *ex vivo* culture period and then added to RIPA lysis buffer (P0013C, Beyotime Biotechnology) containing 1 mM PMSF such that the concentration was 20 mg tissue per 200 μL. Moreover, to extract protein from cultured NP cells, the cells were placed on ice following treatment and washed with ice-cold PBS. Then, RIPA lysis buffer (P0013C, Beyotime Biotechnology) containing 1 mM PMSF was added such that the density was 1×10^6^ cells/100 μL. Next, Western blotting was conducted after collection of the total protein from each of the indicated groups. Briefly, to destroy the 3-dimensional protein structure, the proteins in loading buffer were heated at 100°C for 10 min (Thermo Fisher). Protein electrophoresis (30 μg per lane) was carried out on a 10% SDS-PAGE gel (Beyotime Biotechnology), and the proteins were electroblotted onto nitrocellulose membranes after electrophoresis. The membranes were blocked in 5% nonfat dry milk in Tris-buffered saline with Tween 20 (10 mM Tris-HCl, pH 8.0; 150 mM NaCl; and 0.5% Tween 20) for 2 h and incubated with specific primary antibodies (listed in **Table S4**) for 1 h at 37°C or overnight at 4°C. After washing with PBS three times, horseradish peroxidase-conjugated secondary antibody (diluted 1:2000) was added and incubated for 1 h at room temperature. Membranes were removed from boxes with blunt forceps after washing with PBS at least three times. One millilitre of BeyoECL Plus (P0018S, Beyotime Biotechnology) working solution was added to each membrane for each 10 cm^2^. Bound antibody was visualized using an enhanced chemiluminescence system (Amersham Life Science, Arlington Heights, IL, USA).

### Immunohistochemistry

Mouse samples, rat IVD tissues and human NP samples were decalcified, dehydrated, cleared with dimethylbenzene after fixation in 4% paraformaldehyde, and specimens were embedded in paraffin. Each slice was cut into 5-μm thick sections, which were pretreated with antigen retrieval buffer (enzymatic digestion) (AR0022; Boster Biological Technology, Wuhan, China) for 30 min at 37°C. After blocking in goat serum for 30 min at room temperature, serial slices were incubated with primary antibodies (listed in **Table S5**) at 4°C overnight, followed by incubation with a horseradish peroxidase-conjugated secondary antibody for 60 min at room temperature. Detection was performed by using the VECTASTAIN Elite ABC kit (Vector, Burlingame, CA, USA), and incubation with 0.5 mg/mL 3,3'-diaminobenzidine in 50 mM Tris-Cl (Sigma Aldrich) was used for visualization. Then, the slides were counterstained with 1% haematoxylin.

### Histological staining

Samples originating from *ex vivo*-cultured murine IVD tissues and human samples were dissected and fixed in 4% paraformaldehyde for 3 days. After decalcification in 10% EDTA (pH 7.2-7.4), the samples were processed, embedded in paraffin and cut into 5-μm sections using a microtome. Standard H&E staining was used to examine tissue histology. Briefly, the sections were stained with a haematoxylin solution for 5 min and incubated in 1% acid ethanol (1% HCl in 70% ethanol). The sections were washed with pure water. Then, the sections were stained with an eosin solution for 3 min, dehydrated with graded alcohol and cleared in xylene. Safranin O and fast green staining was performed to determine changes in proteoglycans. The sections were stained with a Safranin O staining kit (G1371, Solarbio) according to the manufacturer's recommended procedure.

### Immunofluorescence staining

Immunofluorescence staining of NP cells and the indicated IVD tissues was performed with anti-cortistatin (diluted 1:150, sc-393108, Santa Cruz Biotechnology), anti-TNFα (diluted 1:100, sc-133192, Santa Cruz Biotechnology), anti-Col 2 (diluted 1:100, sc-52658, Santa Cruz Biotechnology), anti-aggrecan (diluted 1:200, 13880-1-AP, Proteintech), anti-Annexin V (diluted 1:100, ab14196, Abcam), anti-NLRP3 (diluted 1:100, ab4207, Abcam), anti-IL-1β (diluted 1:200, ab9722, Abcam), anti-p65 (diluted 1:150, ab86299, Abcam) and anti-MMP13 (diluted 1:100, sc-515284) antibodies. The procedure was conducted as we described previously 35, and images were taken with a fluorescence microscope (Olympus IX51, Japan). The immunofluorescence signal intensities were quantified with ImageJ software 41.

### Histological assessment

According to previously published articles, we conducted histological grading of the nucleus pulposus (NP) samples 42,43. This classification system was based on an extensive semi-quantitative histological analysis of the NP tissue. Histological scoring was based mainly on proteoglycan loss. A higher score indicated more severe disc degeneration. To assess the differences in immunohistochemistry staining, we determined the percentage of positive cells (brown) for each group and compared these values as previously reported 44,45. All histological assessments were conducted in a blinded manner.

### Enzyme-linked immunosorbent assay (ELISA)

The conditioned media of both cultured NP cells and IVD tissues were maintained in a freezer at -20°C until measurements. The levels of IL-1β were assayed through ELISA by using a commercial kit (eBioscience, Frankfurt, Germany) according to the manufacturer's instructions.

### Transmission electron microscopy (TEM)

Both human and murine NP cells were collected by trypsinization, transferred into 2 mL centrifuge tubes and fixed with fixation solution (Servicebio, G1102) for 2 h at 4°C. The cells were post-fixed in 1% osmium tetroxide in 0.1 M phosphate buffer (pH 7.4) for 2 hours at room temperature (20°C). Afterwards, the NP cells were dehydrated in a graded ethanol series (50%, 70%, 80%, 90%, 95%, 100%, 100%) for 15 minutes for each solution and infiltrated with propylene oxide to embedding medium overnight. Ultrathin sections (50 nm) were obtained using an EM UC7 ultramicrotome (Leica), post-stained with uranyl acetate and lead citrate, and visualized using a transmission electron microscope (HT7700; Hitachi, Tokyo, Japan) 46.

### MitoTracker assay

MitoTracker staining was performed to visualize mitochondria in the primary human NP cells of each indicated group. The procedure was performed in accordance with the instructions of the MitoTracker assay kit (C1049; MitoTracker Red CMXRos, Beyotime Biotechnology) with minor modifications 41. NP cell nuclei were not stained, while the cytoskeleton was stained with phalloidin (green).

### JC-1 assay

To detect the mitochondrial membrane potential in this study, a JC-1 assay kit was used (C2006; Beyotime Biotechnology). Based on the manufacturer's instructions, primary human NP cells from each indicated group in 24-well plates were stained with a JC-1 staining solution at 37°C for 20 minutes while protected from light 47. Then, each well in the plate was washed twice with 1× JC-1 staining buffer, and the fluorescence intensity was measured with an LSM780 laser scanning confocal microscope (ZEISS, Germany) system. The red to green fluorescence ratio reflected changes in the mitochondrial membrane potential.

### ATP production assay

To quantify intracellular ATP production, an ATP bioluminescence assay kit (S0026; Beyotime Biotechnology) was used in this study. All procedures were performed in accordance with the manufacturer's instructions 48.

### Reactive oxygen species assay

To detect intracellular reactive oxygen species (ROS), we used an ROS assay kit (S0033; Beyotime Biotechnology). All the procedures were performed in accordance with the manufacturer's instructions. Briefly, after washing twice with sterile PBS, NP cells were stained with 10 μM DCFDA at 37°C for 20 minutes in the dark and mixed every 4 minutes. Then, the NP cells were washed with serum-free culture medium three times to diminish interference from excess DCFDA 49. The DCFDA fluorescence intensity in each group was measured with an LSM780 laser scanning confocal microscope (ZEISS, Germany) system.

### Seahorse respiration assay

Normal cultured murine NP cells isolated from 6-month-old WT and CST^-/-^ mice were seeded at a density of 20,000 cells/well in a 24-well XF Analyzer plate (Seahorse Bioscience). The extracellular acidification rate (ECAR) and oxygen consumption rate (OCR) were measured using a Seahorse XFe 96 Analyzer (Seahorse Bioscience, Agilent). All measurements from this assay were normalized to the total protein content 50.

### Micro-CT

The scanning protocol included an isometric resolution of 15 μm, with X-ray energy settings of 70 kV and 200 μA. The microstructure of the vertebrae was measured using a Scanco μCT50 scanner (Scanco Medical AG, Bassersdorf, Switzerland). Prior to histological processing, samples were fixed in paraformaldehyde and used for micro-CT. The scanned images from each group were evaluated at the same threshold to allow 3-dimensional structural reconstruction of each sample.

### GAG assay

GAG release from cultured human NP tissue was assessed using DMMB dye (Polysciences, Warrington, PA, USA). All the procedures involved in this assay were performed according to the manufacturer's guidelines, and the GAG assay was performed as previously reported 51. The data are shown as the mean concentration of GAGs released into the conditioned media from three explants (treated in separate wells).

### Flow cytometry

Briefly, cultured human NP cells from each indicated group and murine NP cells from 6-month-old WT and CST^-/-^ mice were detected by flow cytometry. Cells were stained with propidium iodide (PI) and Annexin V-FITC for 15 min at room temperature in the dark in accordance with the protocol of the BD Pharmingen FITC Annexin V Apoptosis Detection Kit I (BD Biosciences, USA). Cell apoptosis was assayed with a CytoFLEX S flow cytometer (Beckman Coulter, USA). The data obtained from this assay were analysed with FlowJo software.

### TUNEL staining

To examine the apoptosis of human NP cells in each indicated group, cells were stained with a One Step TUNEL Apoptosis Assay Kit (C1088; Beyotime Biotechnology). All the procedures were performed according to the manufacturer's instructions 52.

### Statistical analysis

Total data acquisition was conducted in a blinded manner. For comparisons of various treatment groups, the unpaired Mann-Whitney *t*-test, paired Student's *t*-test, and 1-way or 2-way ANOVA (when appropriate) were performed. For ANOVA, Bonferroni *post hoc* analysis was used to compare treatment groups. Fisher's LSD was used to analyse comparisons between multiple groups and the control group. All statistical analyses were performed with GraphPad Prism software (version 7.0; GraphPad Inc., La Jolla, CA, USA). Data are expressed as the mean ± standard deviation (SD). Statistical significance was indicated when *P<*0.05.

## Results

### CST expression was diminished in degenerative NP cells

**Figure 1:**
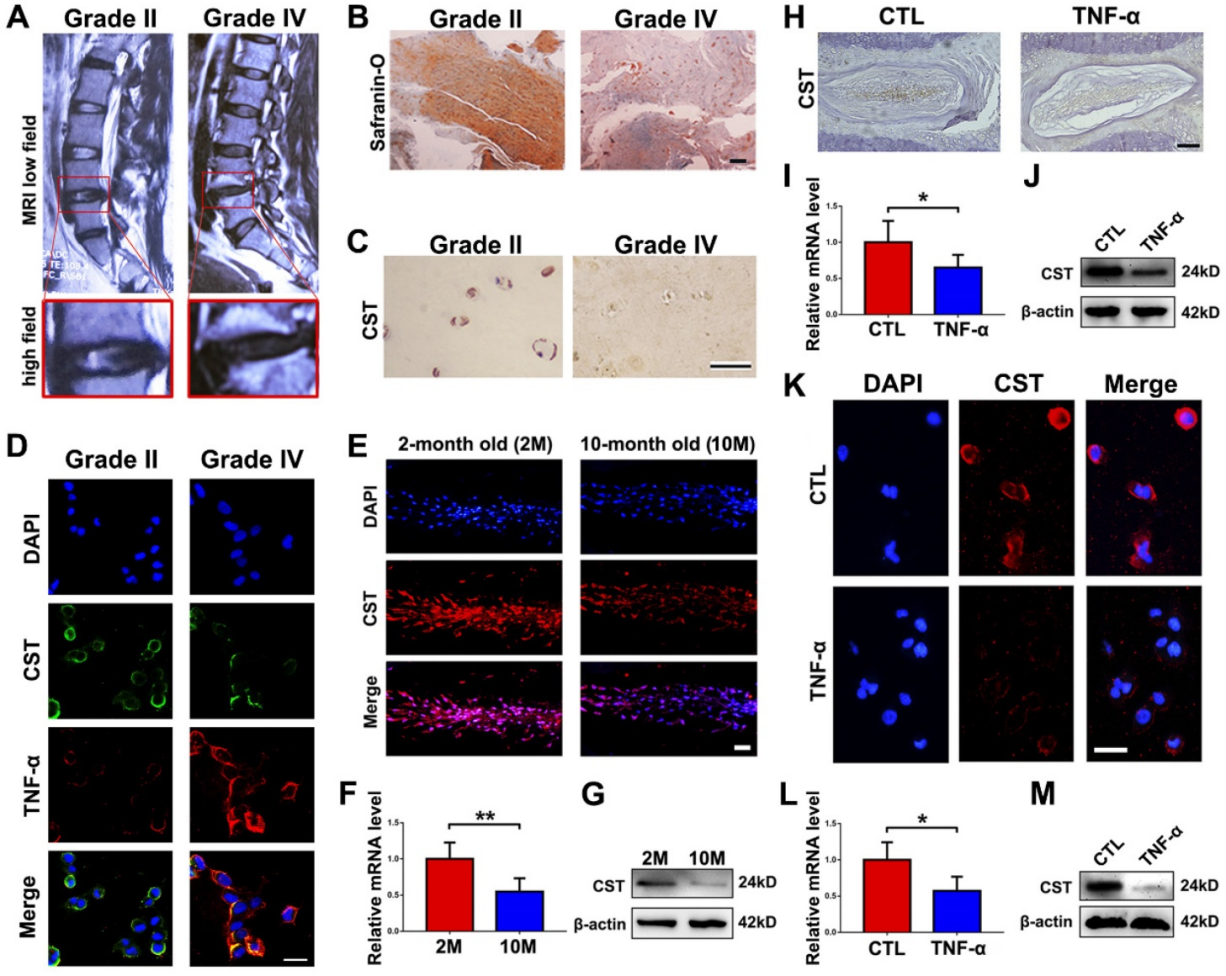
** CST expression was diminished in degenerative NP cells. (A)** Representative MRI images of the lumbar spine from patients with Pfirrmann grade II (n=10) or grade IV (n=7) IDD. The lower panels show pictures of L4/5 segments at a high magnification. **(B)** NP tissues were separately isolated from patients with Pfirrmann grade II or grade IV IDD and subjected to Safranin O staining. Scale bar, 150 µm. **(C)** The CST expression level was diminished in degenerative human NP tissues (grade IV), as detected by immunohistochemistry. Scale bar, 50 µm. **(D)** Human NP cells were isolated from patients with Pfirrmann grade II IDD, and CST expression levels were reduced while TNF-α levels were enhanced, as assayed by cell immunostaining. Nuclei were stained with DAPI. Scale bar, 50 µm. **(E)** Expression of CST in the NP tissues of 2-month-old and 10-month-old WT mice was assessed through immunofluorescence (n=5). Scale bar, 100 µm. **(F)** RNA levels of CST were downregulated in 10-month-old mouse IVD tissues (n=5), as measured by real-time PCR. Total mRNA was collected from 2- and 10-month-old mouse IVD tissues, and real-time PCR was performed. Normalized values were calibrated against the 2-month-old mouse group and given a value of 1.** (G)** Expression of CST in 2- and 10-month-old mouse IVD tissues, as assayed through Western blot analysis (n=5). **(H)** Expression of CST in NP tissues was diminished upon stimulation with TNF-α, as detected by immunohistochemistry (n=5). NP tissues were isolated from 2-month-old WT mice and stimulated with or without 10 ng/ml TNF-α for 24 h. Scale bar, 150 µm. **(I)** IVD tissues from WT mice were induced by TNF-α, and RNA levels of CST were measured through real-time PCR. Normalized values were calibrated against the control (CTL) group and given a value of 1.** (J)** TNF-α induction reduced CST expression in murine IVD, as measured through Western blot analysis (n=5). **(K)** Expression of CST in primary human NP cells following stimulation with TNF-α was detected through cell immunostaining (n=5). Nuclei were stained with DAPI. Scale bar, 100 µm. **(L)** TNF-α reduced CST in primary human NP cells, as measured by real-time PCR (n=5). Normalized values were calibrated against the control (CTL) group and given a value of 1.** (M)** Expression of CST in TNF-α-induced human NP cells, as assessed by Western blot analysis (n=5). *p<0.05 and **p<0.01 vs. the control group. Data are presented as the mean ± SD.

To investigate the expression level of CST in IVD degeneration, NP tissues from patients with Pfirrmann grade II (n=10) or grade IV (n=7) IVD degeneration according to MRI (**Figure 1A**) were collected during surgeries as described in the **Materials and Methods** section, and Safranin O staining and immunohistochemistry for CST were performed. As shown in **Figures 1B-C and Figure S1A** and **S1D**, the expression level of CST was decreased in degenerative NP tissues (grade IV). Moreover, NP cells from each group were isolated and cultured for 24 h, followed by cell immunostaining. **Figure 1D** and **Figure S1B-C** show that the expression of CST was negatively associated with that of TNF-α. In addition, NP tissues were collected from 2-month-old and 10-month-old WT mice (n=5 for each group), and **Figure 1E-G** shows that the expression level of CST was decreased in the 10-month-old mouse NP tissues compared to the 2-month-old mouse NP samples. Furthermore, NP tissues were isolated from 2-month-old WT mice and primary NP cells from patients and stimulated with or without 10 ng/mL TNF-α for 24 h. Immunohistochemistry/immunofluorescence, real-time PCR and Western blot analysis revealed a reduction in CST levels in murine NP tissue (**Figure 1H-J** and **Figure S1E**) and human NP cells (**Figure 1K-M** and**Figure S1F**) following stimulation with TNF-α.

### CST deficiency led to accelerated IVD degeneration

**Figure 2:**
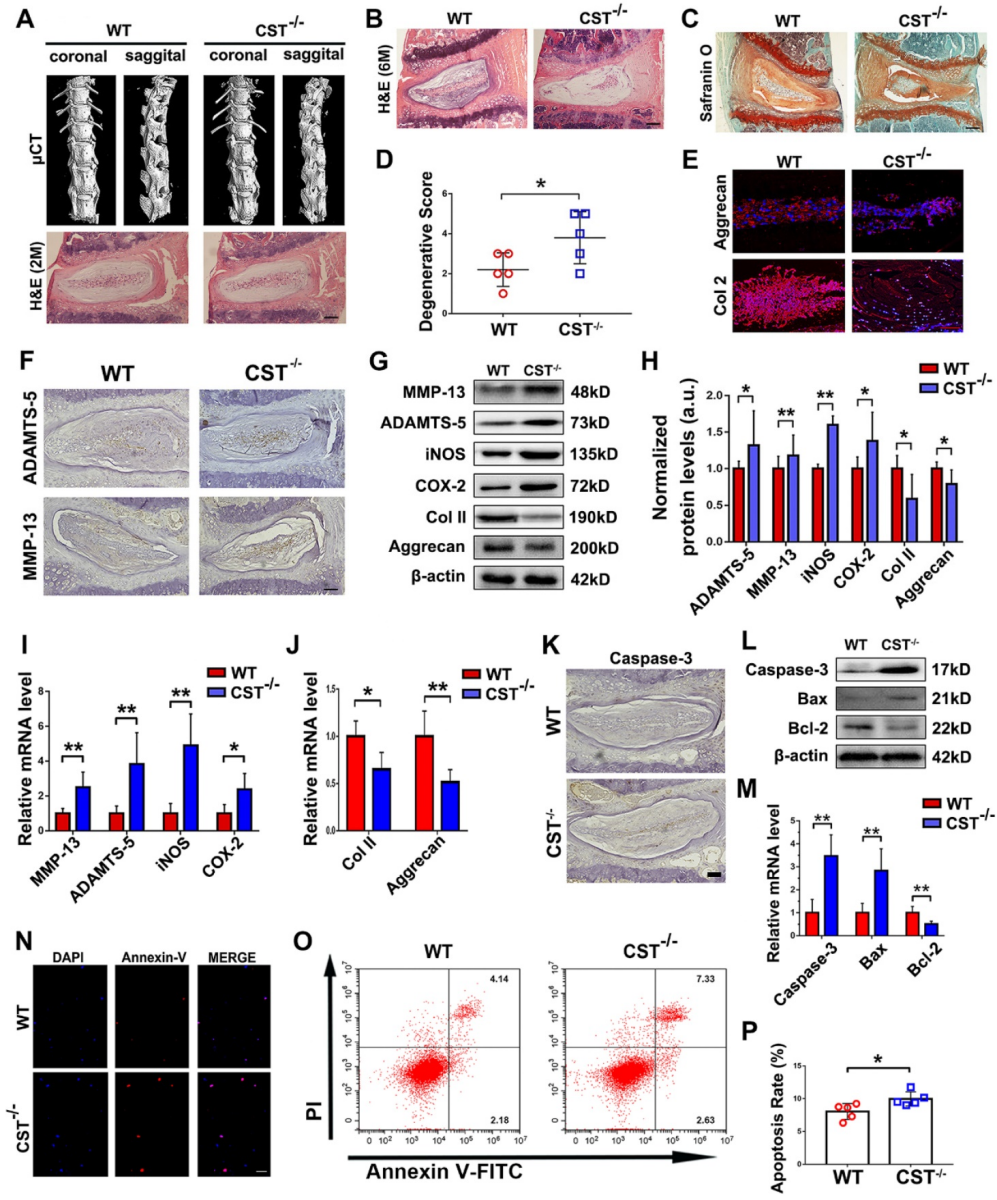
** CST deficiency contributed to accelerated IVD degeneration in mice. (A)** Representative µCT images of the spine and H&E staining of the IVDs from 2-month-old WT and CST^-/-^ mice revealed no overt degeneration in CST^-/-^ mice (n=7). Scale bar, 150 µm.** (B)** H&E staining of 6-month-old WT and CST^-/-^ mice (n=5). Scale bar, 150 µm.** (C-D)** Safranin O staining of 6-month-old CST^-/-^ mouse IVD tissues and appended degenerative scores indicated accelerated IVD degeneration compared with that in the WT littermates (n=5). Scale bar, 150 µm.** (E)** Immunofluorescence to detect Aggrecan and Col 2 in IVD tissues from 6-month-old WT and CST^-/-^ mice (n=5). Scale bar, 150 µm. **(F)** Immunohistochemistry to detect ADAMTS-5 and MMP-13 in IVD tissues from 6-month-old WT and CST^-/-^ mice (n=5). Scale bar, 150 µm.** (G-H)** Western blot analysis of catabolic markers (MMP-13 and ADAMTS-5), inflammatory molecules (iNOS and COX-2) and anabolic markers (Col 2 and Aggrecan) from 6-month-old WT and CST^-/-^ mice (n=5) and analysis of the normalized protein levels in each group. **(I-J)** Real-time PCR to detect catabolic markers (MMP-13 and ADAMTS-5), inflammatory molecules (iNOS and COX-2) and anabolic markers (Col 2 and Aggrecan) from 6-month-old WT and CST^-/-^ mice (n=5). Total mRNA was collected from each indicated group, and real-time PCR was performed. Normalized values were calibrated against the wild-type (WT) group and given a value of 1.** (K)** Expression of caspase-3 in IVD tissues was elevated in 6-month-old CST^-/-^ mice compared with WT littermates, as detected by immunohistochemistry (n=5). Scale bar, 150 µm. **(L)** Expression of caspase-3, bax and bcl-2 was detected by Western blot analysis (n=5). Total protein was collected from the IVD tissues of 6-month-old WT and CST^-/-^ mice, and Western blot analysis was performed. **(M)** Expression of caspase-3, bax and bcl-2 was examined by real-time PCR (n=5). Normalized values were calibrated against the wild-type (WT) group and given a value of 1.** (N)** Immunofluorescence to detect Annexin-V in NP cells isolated from 6-month-old WT and CST^-/-^ mice (n=5). Nuclei were stained with DAPI. Scale bar, 100 µm. **(O)** Quantification of NP cell apoptosis in the WT and CST^-/-^ mice, as detected by flow cytometry (n=5). **(P)** Apoptosis rates in the two groups. *p<0.05 and **p<0.01 vs. the WT group. Data are presented as the mean ± SD.

To determine whether CST is indispensable for homeostasis of the IVD structure in the ageing process, IVD degeneration in CST^-/-^ mice and WT mice was compared. We previously reported the phenotype of 2-month-old CST^-/-^ mice, which displayed no significant differences compared with their WT littermates 31. In this study, micro-CT of the spine (**Figure 2A** and**Figure S2A-D**), H&E staining of NP tissues (**Figure 2A**), body weight assessment (**Figure S2E**) and the scoring of NP tissue degeneration (**Figure S2F**) revealed no significant difference in the IVD between 2-month-old WT and CST^-/-^ mice. However, H&E staining (**Figure 2B**) and micro-CT (**Figure S2G**) revealed abnormal osteophyte formation and a decrease in the intervertebral space in 6-month-old CST^-/-^ mice compared to their WT littermates (n=5 for each group), which indicated accelerated IVD degeneration. In addition, Safranin O staining, histological scoring to assess degeneration and immunostaining for Aggrecan and Col 2 (n=5 for each group) indicated accelerated extracellular matrix degradation in CST^-/-^ NP tissue through ageing (**Figure 2C-E**). Moreover, immunohistochemistry detected the elevated expression of ADAMTS-5 and MMP-13 (**Figure 2F** and **Figure S2I-J**). IVD tissue was collected from 6-month-old WT and CST^-/-^ mice (n=5 for each group) and subjected to Western blot analysis (**Figure 2G-H**) and real-time PCR (**Figure 2I-J**). The CST^-/-^ group exhibited no CST expression (**Figure S2H**), elevated expression of catabolic markers (ADAMTS-5, MMP-13) and inflammatory molecules (iNOS and COX-2), and decreased production of anabolic markers (Aggrecan and Col 2). Furthermore, immunohistochemistry for Caspase-3 (**Figure 2K** and **Figure S2K**) and Western blotting (**Figure 2L**) and real-time PCR (**Figure 2M**) for Caspase-3, Bcl-2 and Bax were performed. NP tissue from the 6-month-old CST^-/-^ mice displayed enhanced apoptosis compared with that from the WT littermates. Additionally, primary NP cells were isolated from the NP tissues of 6-month-old mice, and CST deficiency enhanced apoptosis, as detected by Annexin-V immunostaining (**Figure 2N** and**Figure S2L**) and flow cytometry (**Figure 2O-P**).

### CST knockout exaggerated mitochondrial ROS-dependent activation of the NLRP3 inflammasome

**Figure 3:**
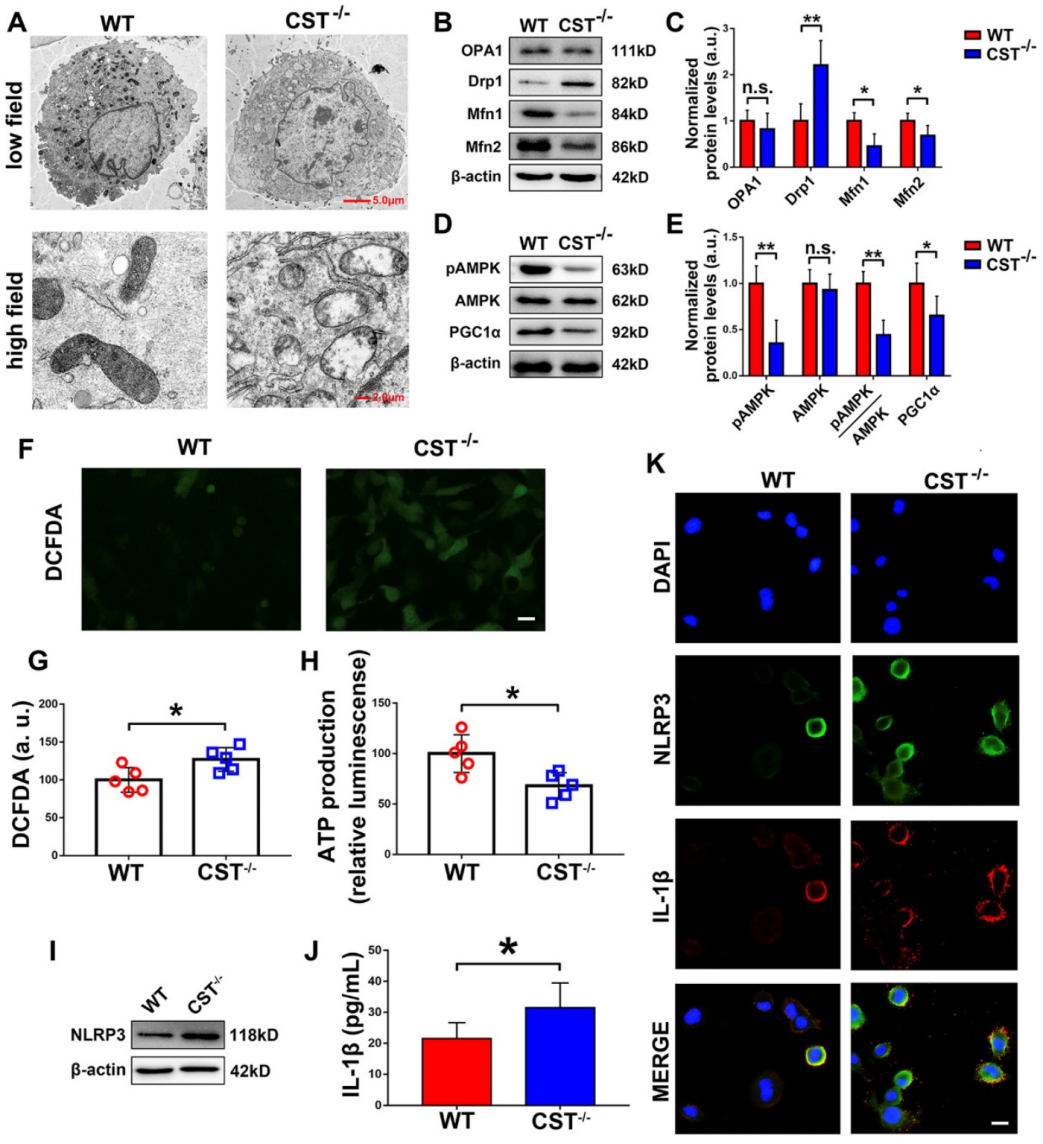
** CST knockout exaggerated mitochondrial ROS-dependent activation of the NLRP3 inflammasome. (A)** Representative TEM images of the mitochondria in NP cells from WT and CST^-/-^ mice (n=5). A low-field image of a whole cell and magnified high-field images of swollen mitochondria are shown. Scale bars, 5 µm (upper panel), 2 µm (lower panel). **(B-C)** Western blot analysis of the mitochondrial morphology-related proteins Drp1, OPA1 and Mfn1/2 and analysis of the normalized protein levels (n=5). Total protein was collected from NP cells isolated from WT and CST^-/-^ mice. **(D-E)** Western blot analysis of pAMPK, AMPK and PGC1α, key regulators of mitochondrial biogenesis and dynamics, and analysis of the normalized protein levels (n=5). Total protein was collected from NP cells isolated from WT and CST^-/-^ mice. **(F-G)** Representative images and quantification of ROS levels in the WT and CST^-/-^ groups (n=5). Scale bar, 20 µm. **(H)** ATP production in the WT and CST^-/-^ groups (n=5). **(I)** Western blot analysis of NLRP3 in the WT and CST^-/-^ groups (n=5). **(J)** The expression of IL-1β in culture media supernatants from the WT and CST^-/-^ groups, as detected by ELISA (n=5). **(K)** Representative images of NLRP3 and IL-1β expression in the WT and CST^-/-^ groups, as assayed by immunofluorescence (n=5). Nuclei were stained with DAPI. Scale bar, 20 µm. *p<0.05 and **p<0.01 vs. the WT control group. n.s., not significant. Data are presented as the mean ± SD.

Mitochondrial dysfunction is closely associated with processes in ageing, including IVD degeneration. To demonstrate the potential interaction between endogenous CST and mitochondrial homeostasis, primary NP cells were isolated from 6-month-old WT and CST^-/-^ mice, and transmission electron microscopy (TEM) was performed. As shown in** Figure 3A**, high-field images of swollen mitochondria in the NP cells from CST^-/-^ mice showed that the mitochondria surrounded the nucleus, indicating accelerated mitochondrial damage, but these changes were not observed in NP cells from WT mice. To further investigate the role of CST in mitochondrial function, the OCR and ECAR were assayed (**Figure S3C-F**), which showed that compared to those of WT mice, the NP cells of CST^-/-^ mice displayed both significantly lower basal respiratory oxygen consumption and reduced glycolytic capacity. In addition, total protein was collected from NP cells, and the mitochondrial morphology-related proteins 41 Drp1, OPA1 and Mfn1/2 (**Figure 3B-C**) and the key regulators of mitochondrial biogenesis and dynamics 41 pAMPK, AMPK and PGC1α (**Figure 3D-E**) were tested through Western blot analysis, which revealed exaggerated mitochondrial damage with CST deficiency. Moreover, ROS levels were detected through the DCFDA assay (**Figure 3F-G**), and ATP production (**Figure 3H**) was measured, which showed that CST knockout exaggerated ROS levels and impaired the function of the respiratory chain. Furthermore, Western blot analysis (**Figure 3I**) of NLRP3, ELISA (**Figure 3J**) to detect IL-1β and cell immunostaining (**Figure 3K** and **Figure S3A-B**) to detect NLRP3 and IL-1β indicated that the production and function of the NLRP3 inflammasome were elevated with CST deficiency.

### Exogenous CST maintained the homeostasis of NP cells under cytokine stimulation and attenuated IVD degeneration in animal models

**Figure 4:**
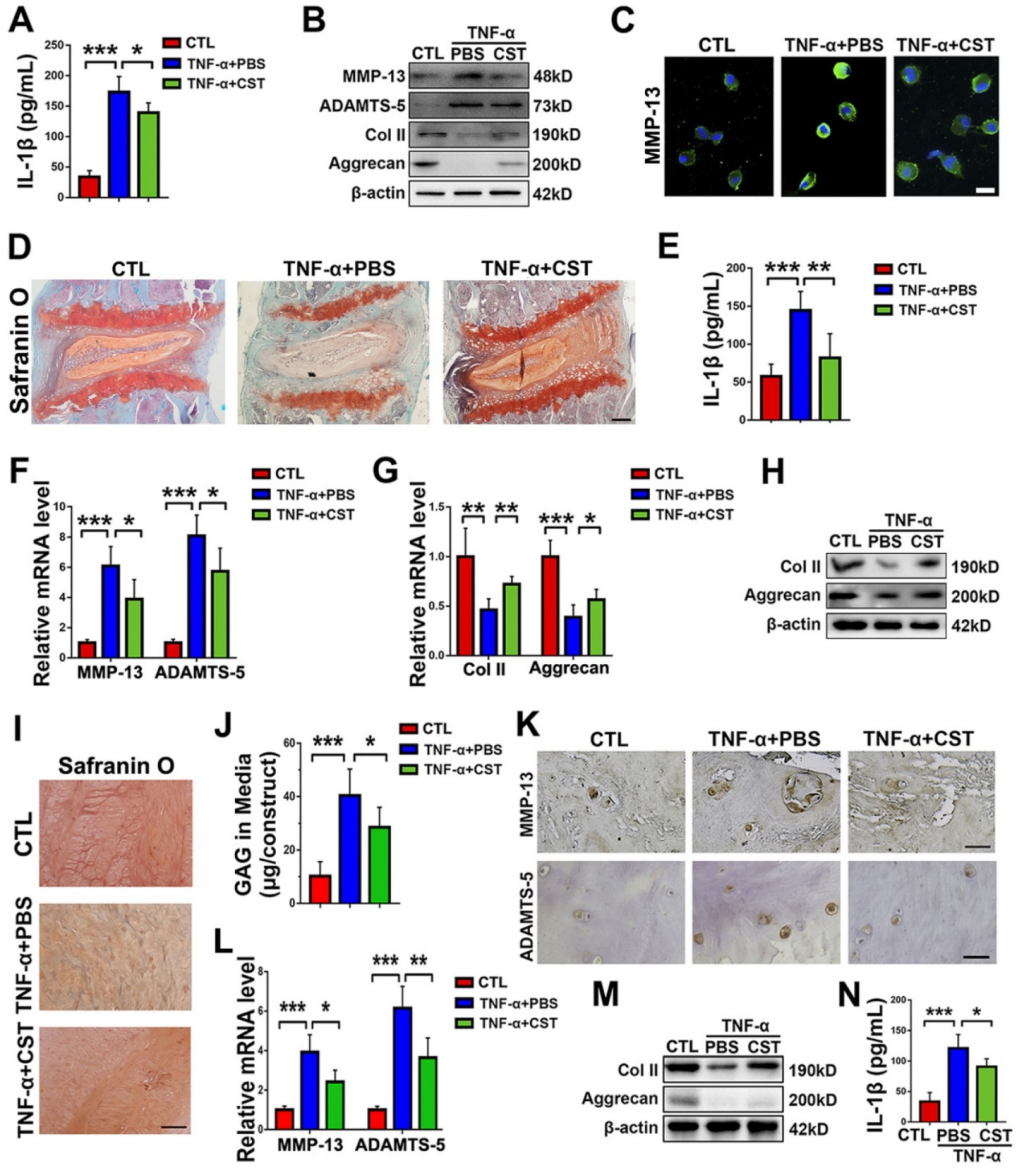
** Exogenous CST restored the homeostasis of cultured NP cells and NP tissues stimulated with TNF-α. (A)** The expression of IL-1β in human NP cell culture media following stimulation with TNF-α with or without additional treatment with exogenous CST, as detected by ELISA (n=5).** (B)** Western blot analysis of MMP-13, ADAMTS-5, Col 2 and Aggrecan in human NP cells (n=5).** (C)** Immunofluorescence of MMP-13 in human NP cells (n=5). Nuclei were stained with DAPI. Scale bar, 20 µm. **(D)** Safranin O staining of *ex vivo*-cultured murine IVD tissues (n=5). Murine IVD tissues were cultured under TNF-α stimulation in the presence or absence of CST. Scale bar, 150 µm. **(E)** Expression of IL-1β in the culture media of murine IVD tissues (n=5). **(F-G)** The mRNA expression of MMP-13, ADAMTS-5, Col 2 and Aggrecan, as measured by real-time PCR. Total mRNA was collected from the murine IVD tissues of each indicated group, and real-time PCR was performed (n=5). Normalized values were calibrated against the control group and given a value of 1.** (H)** Western blot analysis of Col 2 and Aggrecan in murine IVD tissues (n=5). **(I)** Representative images showing Safranin O staining of *ex vivo*-cultured human IVD tissues (n=5). Human IVD tissues were cultured under TNF-α stimulation in the presence or absence of CST. Scale bar, 100 µm. **(J)** The release of GAG in the culture media of each group of *ex vivo*-cultured human IVD tissues (n=5). **(K)** Immunohistochemistry to detect MMP-13 and ADAMTS-5 in *ex vivo*-cultured human IVD tissues (n=5). Scale bar, 100 µm. **(L)** Relative mRNA expression of MMP-13 and ADAMTS-5 in *ex vivo*-cultured human IVD tissues, as measured by real-time PCR (n=5). **(M)** Western blot analysis of Col 2 and Aggrecan in *ex vivo*-cultured human IVD tissues (n=5). **(N)** The expression of IL-1β in the culture media of groups of *ex vivo*-cultured human IVD tissues, as detected by ELISA (n=5). *p<0.05, **p<0.01 and ***p<0.001 vs. the indicated control group. Data are presented as the mean ± SD.

To study the role of exogenous CST in IVD degeneration, TNF-α stimulation was used to induce IVD degeneration 16. Human primary NP cells were isolated and stimulated with 10 ng/mL TNF-α for 24 h, with or without treatment with recombinant CST peptide (50 μg/mL). Thereafter, the conditioned media were collected, and IL-1β expression was assayed through ELISA. As shown in **Figure 4A**, TNF-α-induced secretion of IL-1β was suppressed by exogenous CST treatment. In addition, total protein was collected, and Western blot analysis revealed that CST inhibited the disordered expression of the metabolic markers ADAMTS-5, MMP-13, Aggrecan and Col 2 under TNF-α stimulation (**Figure 4B**). NP cell immunostaining was carried out, which showed that CST treatment diminished MMP-13 production induced by TNF-α (**Figure 4C** and**Figure S4A**). In addition, human NP cells were stimulated with 10 ng/mL IL-1β for 24 h with or without treatment with recombinant CST peptide (50 μg/mL). CST was found to inhibit the production of ADAMTS-5 and MMP-13 induced by IL-1β (**Figure S4B**). Moreover, NP tissues from WT mice were cultured under TNF-α stimulation *ex vivo* with or without treatment with CST. Safranin O staining (**Figure 4D**) indicated that CST alleviated the loss of proteoglycans, and ELISA revealed that CST diminished the secretion of IL-1β (**Figure 4E**). Furthermore, real-time PCR (**Figure 4F-G**) and Western blotting (**Figure 4H**) showed that disorganized expression of metabolic biomarkers triggered by TNF-α was protected against by CST. Human NP tissues (n=5 for each group) were isolated from patients and cultured under 10 ng/mL TNF-α stimulation in the presence or absence of exogenous CST (50 μg/mL). Safranin O staining and the detection of released GAG indicated that CST attenuated TNF-α-mediated induction of proteoglycan loss (**Figure 4I-J**). Elevated expression of ADMATS-5 and MMP-13 mediated by TNF-α was detected by immunohistochemistry (**Figure 4K** and**Figure S4C-D**) and real-time PCR (**Figure 4L**), but this increase in expression was suppressed by exogenous CST. In addition, total protein was collected (**Figure 4M**), and conditioned medium (**Figure 4N**) was isolated from human NP tissue, followed by Western blot analysis of anabolic biomarkers and ELISA of IL-1β to test the protective role of CST in cytokine-induced NP degeneration.

To further study the role of CST in IVD degeneration *in vivo*, a rat tail needle puncture model of IDD was established 14,40; the rats were or were not treated with exogenous CST to investigate whether CST plays a protective role against IDD *in vivo*. The tails of the rats were collected four weeks post-puncture, after which H&E staining and immunohistochemistry were performed, which revealed that CST suppressed IVD degeneration and inhibited the loss of Col II (**Figure S4E-G**). In addition, the caudal vertebrae of the rats were assayed by X-ray, and the corresponding disc height index (DHI) was measured. Exogenous CST attenuated osteophyte formation and increased the intervertebral space in this model of IVD degeneration (**Figure S4H-I**).

### CST treatment suppressed mitochondrial ROS-dependent activation of the NLRP3 inflammasome in NP cells

**Figure 5:**
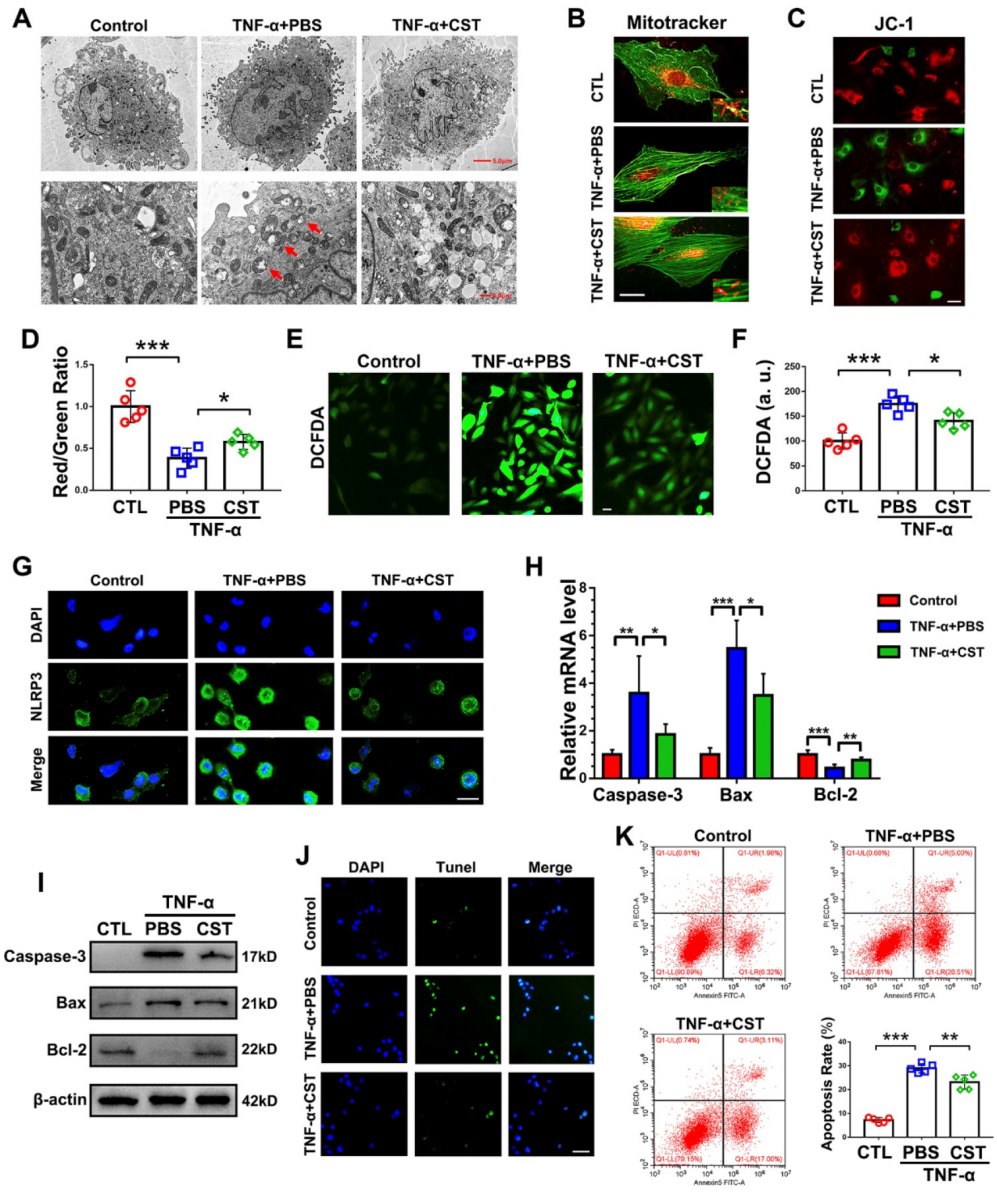
** CST treatment suppressed mitochondrial ROS-dependent activation of the NLRP3 inflammasome in NP cells. (A)** Representative TEM images of mitochondria in human NP cells from each indicated group (n=5). Human NP cells were cultured under TNF-α stimulation in the presence or absence of CST. A low-field image of a whole cell and magnified high-field images are shown. Swollen mitochondria in TNF-α-stimulated NP cells are indicated by red arrows. Scale bar, 5 µm (upper panel), 2 µm (lower panel). **(B)** Representative fluorescence images of mitochondria in NP cells (n=5). Cells were counterstained with phalloidin (green). Scale bar, 10 µm. **(C)** JC-1 assay of NP cells in each indicated group (n=5). Scale bar, 20 µm. **(D)** The red: green fluorescence ratio used for quantification via JC-1 assay (n=5). **(E-F)** Representative images and quantification of ROS levels in human NP cells in each indicated group (n=5). Scale bar, 20 µm. **(G)** Representative immunofluorescence images showing NLRP3 in human NP cells (n=5). Scale bar, 20 µm. **(H)** Relative mRNA expression of caspase-3, bax and bcl-2 in the human NP cells of each indicated group, as measured by real-time PCR (n=5). **(I)** Western blot analysis of caspase-3, bax and bcl-2 in the human NP cells of each indicated group (n=5). **(J)** TUNEL staining of the human NP cells of each indicated group (n=5). Nuclei were stained with DAPI. Scale bar, 100 µm. **(K)** Quantification of NP cell apoptosis by flow cytometry (n=5). *p<0.05, **p<0.01 and ***p<0.001 vs. the indicated control group. Data are presented as the mean ± SD.

To determine whether the protective role of exogenous CST is associated with mitochondrial ROS and activation of the NLRP3 inflammasome, primary human NP cells were cultured under stimulation with 10 ng/mL TNF-α with or without treatment with recombinant CST (50 μg/mL) and examined by TEM. As shown in **Figure 5A**, swollen mitochondria were detected following the administration of TNF-α, but CST treatment attenuated mitochondrial damage induced by TNF-α. In addition, a MitoTracker assay was performed to detect the mitochondrial morphology 53, which showed that mitochondrial fission induced by TNF-α was reversed by exogenous CST (**Figure 5B**). The mitochondrial membrane potential was measured through the JC-1 assay, the results of which (**Figure 5C-D)** indicated that TNF-α-mediated mitochondrial damage was reversed by CST. In this study, ROS levels were measured through the DCFDA assay. As revealed in **Figure 5E-F**, TNF-α elevated the ROS levels in NP cells, while exogenous CST inhibited this increase in mitochondrial ROS. Moreover, the expression of NLRP3 was detected through cell immunostaining, which showed that the enhanced fluorescence signal for NLRP3 in the TNF-α-treated group was decreased by CST treatment (**Figure 5G** and**Figure S5**). To assess the mitochondrial apoptotic pathway, levels of the associated biomarkers Caspase-3, Bax and Bcl-2 in NP cells were tested through real-time PCR (**Figure 5H**) and Western blot analysis (**Figure 5I**), which showed that the increased expression of these molecules induced by TNF-α was remarkably maintained by exogenous CST. Furthermore, TUNEL staining (**Figure 5J**) and flow cytometry (**Figure 5K**) were performed to detect apoptosis, which showed that CST antagonized TNF-α-induced apoptosis in NP cells.

### CST protects against IVD degeneration through regulating the NF-κB signalling pathway, suppressing mitochondrial ROS generation and inhibiting NLRP3 activation

**Figure 6:**
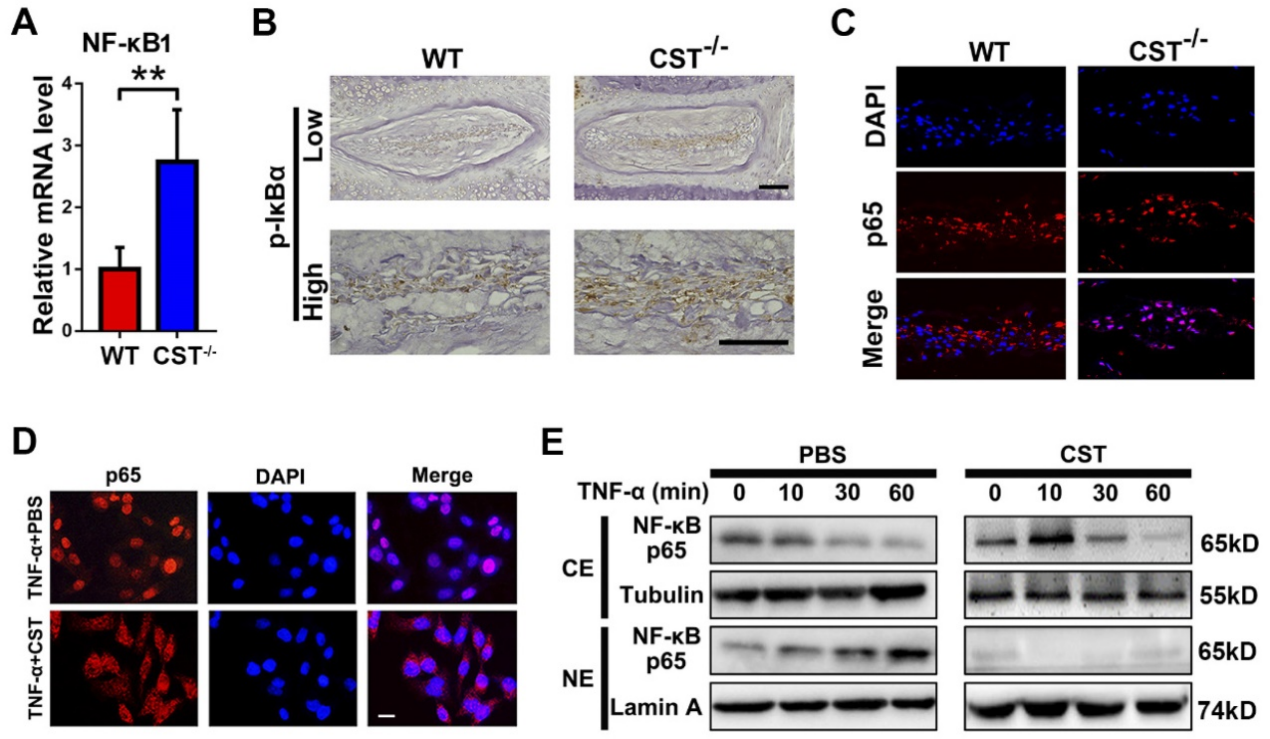
** CST protected against IVD degeneration through regulating the NF-κB signalling pathway. (A)** Relative mRNA expression of NF-κB1 in IVD tissues from 6-month-old WT and CST^-/-^ mice, as measured by real-time PCR. Normalized values were calibrated against the wild-type (WT) group and given a value of 1 (n=5). **(B)** Immunohistochemistry to detect pIκBα in IVD tissues from 6-month-old WT and CST^-/-^ mice (n=5). Scale bar, 150 µm. **(C)** Nuclear translocation of p65 in NP tissue was enhanced in CST^-/-^ mice, as detected by immunofluorescence (n=5). Scale bar, 100 µm. **(D)** Representative immunofluorescence images for p65 in the cultured human NP cells of each indicated group (n=5). Nuclei were stained with DAPI. Scale bar, 100 µm. **(E)** Exogenous CST antagonized the nuclear translocation of NF-κB p65 in human NP cells, as assayed by Western blot analysis. Tubulin and Lamin A are shown as loading controls (n=5). **p<0.01 vs. the indicated control group. Data are presented as the mean ± SD.

As CST antagonizes the NF-κB signalling pathway 31 and the NF-κB signalling pathway is closely associated with the function of TNF-α and mitochondrial ROS-dependent NLRP3 inflammasome activation 22,23, we aimed to determine whether CST interacts with the TNF-α/NF-κB signalling pathway in IVD degeneration. NP tissues were collected from 6-month-old WT and CST^-/-^ mice, and real-time PCR to detect NF-κB1 (**Figure 6A**), immunohistochemistry (**Figure 6B**) to detect p-IκBα and immunofluorescence staining to detect P65 (**Figure 6C** and**Figure S6**) were performed. CST deficiency enhanced the activity of the NF-κB signalling pathway. Next, human NP cells were collected, and immunofluorescence staining and Western blotting of these cells showed that the TNF-α-induced nuclear translocation of p65 was abolished by treatment with exogenous CST (**Figure 6D-E**).

**Figure 7:**
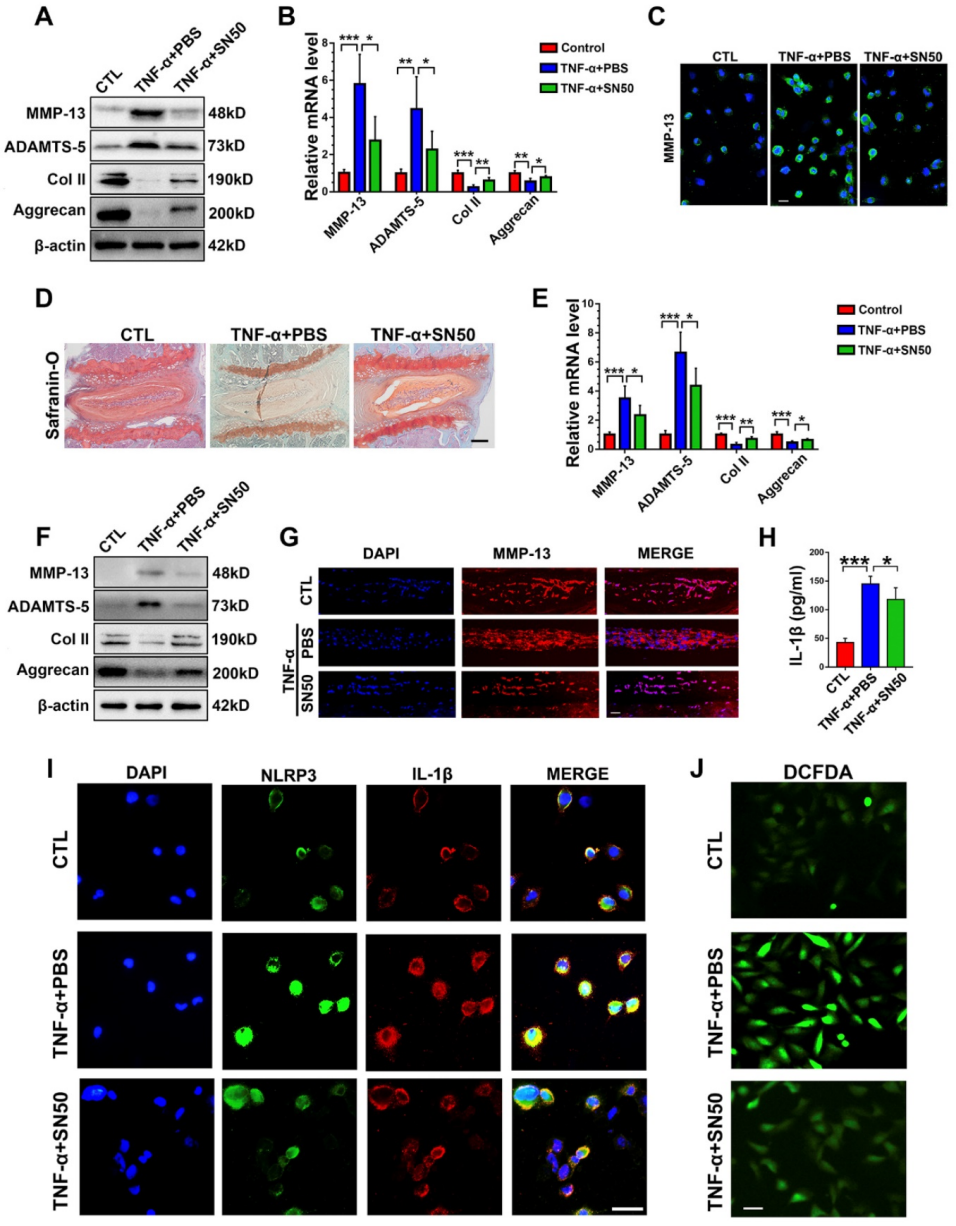
** CST mimics the role of SN50 in inhibition of the NF-κB signalling pathway. (A)** Western blot analysis of MMP-13, ADAMTS-5, Col 2 and Aggrecan in CST^-/-^ NP cells (n=5). Primary CST^-/-^ NP cells were cultured under TNF-α, stimulation with or without additional treatment with SN50, followed by Western blot analysis. **(B)** Relative mRNA expression of MMP-13, ADAMTS-5, Col 2 and Aggrecan in murine NP cells from CST^-/-^ mice, as measured by real-time PCR (n=5). **(C)** Immunofluorescence of MMP-13 in murine NP cells from CST^-/-^ mice (n=5). Scale bar, 20 µm. **(D)** Safranin O staining of *ex vivo*-cultured CST^-/-^ murine IVD tissues in each indicated group (n=5). Scale bar, 150 µm. **(E)** Relative mRNA expression of MMP-13, ADAMTS-5, Col 2 and Aggrecan in *ex vivo*-cultured CST^-/-^ murine IVD tissues, as measured by real-time PCR (n=5). **(F)** Western blot analysis of MMP-13, ADAMTS-5, Col 2 and Aggrecan in *ex vivo*-cultured CST^-/-^ murine IVD tissues of each indicated group (n=5). **(G)** Immunofluorescence of MMP-13 in the *ex vivo*-cultured CST^-/-^ murine IVD tissues of each indicated group. (n=5). Scale bar, 100 µm. **(H)** Expression of IL-1β in the culture media of groups of murine CST^-/-^ NP cells, as detected by ELISA (n=5).** (I)** Representative images of NLRP3 and IL-1β immunofluorescence in the murine CST^-/-^ NP cells of each indicated group (n=5). Scale bar, 20 µm.** (J)** Representative images of ROS levels in the murine CST^-/-^ NP cells of each indicated group (n=5). Scale bar, 20 µm. *p<0.05, **p<0.01 and ***p<0.001 vs. the indicated control group. Data are presented as the mean ± SD.

To determine whether blockade of NF-κB activation would alleviate cytokine-induced NP degeneration in CST deficiency, primary NP cells were collected from CST^-/-^ mice and cultured under 10 ng/mL TNF-α stimulation with or without treatment with the NF-κB inhibitor SN50 for 24 h. Thereafter, Western blot (**Figure 7A**), real-time PCR (**Figure 7B**) and cell immunostaining (**Figure 7C** and**Figure S7A**) for the indicated metabolic markers were performed, which showed that inhibition of NF-κB signalling reversed the TNF-α-mediated disturbance in metabolic homeostasis in CST^-/-^ NP cells. Moreover, CST^-/-^ murine NP tissues were isolated, and *ex vivo* experiments indicated that SN50 attenuated TNF-α-induced disordered expression of metabolic biomarkers and destruction of the IVD, as revealed by safranin O staining (**Figure 7D**), real-time PCR (**Figure 7E**), Western blot analysis (**Figure 7F**) and an immunofluorescence assay (**Figure 7G**). To study the participation of the NF-κB signalling pathway in activation of the NLRP3 inflammasome, primary CST^-/-^ NP cells were stimulated with TNF-α with or without SN50 treatment. Thereafter, cell immunostaining for NLRP3 and IL-1β (**Figure 7I** and**Figure S7B-C**), an ROS assay (**Figure 7J**) and ELISA to detect IL-1β (**Figure 7H**) were performed, which indicated that antagonization of the NF-κB signalling pathway markedly inhibited activation of the NLRP3 inflammasome in CST deficiency.

To further study the interaction between the NF-κB signalling pathway and activation of the NLRP3 inflammasome in CST^-/-^ NP tissue, IVD tissues were isolated from 6-month-old CST^-/-^ mice and then cultured *ex vivo* in the presence of PBS or SN50 for 24 h. As presented in **Figure S7D-E**, SN50 effectively inhibited the activation of NLRP3, as detected by immunohistochemistry and Western blot analysis. Furthermore, the supernatant of the culture medium for each indicated group was collected, and ELISA was then performed. SN50 significantly downregulated the expression of IL-1β in CST deficiency (**Figure S7F**).

To investigate whether the requirement of CST for NP homeostasis is associated with the regulation of mitochondrial ROS and the NLRP3 inflammasome, primary NP cells were isolated from 2-month CST^-/-^ mice and cultured under 10 ng/mL TNF-α stimulation with or without treatment with the NLRP3 inhibitor MCC950 at 1 μM 54 or the mitochondria-targeted antioxidant peptide Szeto-Schiller 31 (SS-31) at 5 μM 55 for 24 h. MCC950 and SS-31 antagonized the disorganized expression of the NLRP3-downstream molecule IL-1β (**Figures S8A** and **S9A**), inflammatory mediators (**Figures S8B** and **S9B-C**) and metabolic biomarkers (**Figures S8C** and **S9D**) induced by cytokine treatment in NP cells from CST^-/-^ mice.

## Discussion

Intervertebral disc degeneration is a common degenerative disease in the clinic, but much about the underlying mechanisms involved remains unknown 2. Previous reports have shown that disorder in NP cells and the destruction of NP tissue are critical features of IVD degeneration 56. Various studies have focused on the homeostasis of NP cells as a potential treatment for IVD degeneration 3. In addition, several cytokines, especially TNF-α and IL-1β, are commonly utilized to facilitate IVD degeneration and to investigate the therapeutic potential of molecules in IVD degeneration 57,58. In the current study, ageing- and cytokine-induced IVD degeneration models were established, and CST deficiency was found to accelerate the impairment of NP cell and tissue homeostasis, while exogenous CST attenuated this impairment. NP cells and the extracellular matrix, the primary components of the NP structure, become disorganized during the degeneration process 59. Imbalance between anabolism and catabolism is a change characteristic of NP cells during ageing 60. In the present study, compared with their WT littermates, 6-month-old CST^-/-^ mice exhibited enhanced production of the catabolic markers MMP-13 and ADAMTS-5 and diminished levels of the anabolic markers Aggrecan and Col 2, suggesting disordered metabolism in NP cells. Furthermore, CST deficiency exaggerated osteophyte formation and the reduced intervertebral space in the IVD of model animals, while exogenous CST alleviated these changes, suggesting the protective function of CST in IVD ageing 61. Moreover, apoptosis was reported to be elevated in NP cells during IVD degeneration 9,41,62. In this study, the exaggerated apoptosis of NP cells from 6-month-old CST^-/-^ mice was detected, implying accelerated NP ageing in CST deficiency.

Dysfunction of various organelles, especially mitochondria, is closely associated with IVD degeneration 63. Dysfunction and abnormal alteration of the mitochondrial morphology were detected in aged NP cells 7,41. In the current study, primary NP cells from 6-month-old CST^-/-^ mice displayed accelerated cristae fission, exaggerated mitochondrial dysfunction, decreased basal respiratory oxygen consumption and a reduced glycolytic capacity, suggesting the requirement of endogenous CST for mitochondria-associated degeneration. Mitochondria play a key role in mitochondrial ROS and ATP production in NP cells 64. In this study, OPA1, Drp1, Mfn1 and Mfn2, which are morphological and functional biomarkers of mitochondria, were assayed 41, which indicated that CST deficiency triggered mitochondrial dysfunction and disturbed the production of ATP. Moreover, imbalance between Bax and Bcl-2 expression is involved in the mitochondrial apoptotic pathway during IVD degeneration, which induces Caspase-3 expression and facilitates the apoptosis of NP cells 47. In this study, NP cells from CST^-/-^ mice exhibited disordered expression of Bax and Bcl-2 and enhanced expression of Caspase-3, suggesting the association of CST with the mitochondrial apoptotic pathway.

The NLRP3 inflammasome plays a detrimental role in metabolism and apoptosis in NP cells and contributes to IVD degeneration 14. Mitochondria contribute to NLRP3 inflammasome activation through several mechanisms 65,66, including mitochondrial dysfunction and mitochondrial ROS generation 67,68. Here, we found elevated levels of NLRP3 accompanied by accelerated mitochondrial dysfunction in NP cells from CST^-/-^ mice compared with WT mice cells, suggesting enhanced mitochondrial ROS-dependent NLRP3 production. The NLRP3 inflammasome is closely associated with the secretion of active IL-1β 12, and IL-1β is a key player in the progression of cartilage degeneration through various mechanisms, including IVD degeneration 69. In this study, IL-1β secretion was promoted in NP cells from aged CST^-/-^ mice compared with WT mice, suggesting CST deficiency leads to NLRP3 inflammasome activation, consequently enhancing the secretion of IL-1β to cause further damage to NP cells. Furthermore, the NLRP3 inflammasome is activated during different types of cell death, including apoptosis 70, which is consistent with the finding that NP cells from CST^-/-^ mice presented an elevated apoptosis rate, implying the requirement of CST for the maintenance of normal levels of NLRP3 inflammasome-associated apoptosis.

Cytokine induction is a critical risk factor in IVD degeneration 16. TNF-α, a predominant cytokine in IVD degeneration, triggers enhanced inflammation, disordered metabolism and accelerated apoptosis in NP cells 17. Inhibition of TNF-α function or antagonization of the NF-κB signalling pathway attenuated degeneration of the IVD 71. In this study, TNF-α-induced degeneration of NP cells and tissue was reversed by exogenous CST treatment, suggesting the role of exogenous CST in the cytokine-related degeneration of NP cells. TNF-α stimulation is known to cause mitochondrial dysfunction, which facilitates ROS generation and activation of the NLRP3 inflammasome in various cell types 72-74. In the present study, TNF-α-mediated induction of mitochondria-dependent damage to NP cells was suppressed by exogenous CST, which further implied that CST protected against cytokine-induced IVD degeneration. The NF-κB signalling pathway plays a critical role in TNF-α function, which leads to degeneration through several downstream mechanisms 75. Furthermore, the NF-κB signalling pathway is involved in TNF-α-mediated activation of the NLRP3 inflammasome 76, and activation of the NF-κB signalling pathway disturbs the metabolism and apoptosis of NP cells 21, 77. Here, we found that the NF-κB inhibitor SN50 78 suppressed ROS, inhibited activation of the NLRP3 inflammasome and maintained the metabolism of NP cells from CST^-/-^ mice through degeneration, implying that CST interacts with the TNF-α/NF-κB axis, which regulates mitochondrial ROS-dependent NLRP3 inflammasome activation. Moreover, the NLRP3 inhibitor MCC950 and the mitochondria-targeted antioxidant peptide SS-31 effectively attenuated the impaired homeostasis in CST^-/-^ NP cells, suggesting the underlying mechanism of CST in IVD degeneration.

**Figure 8:**
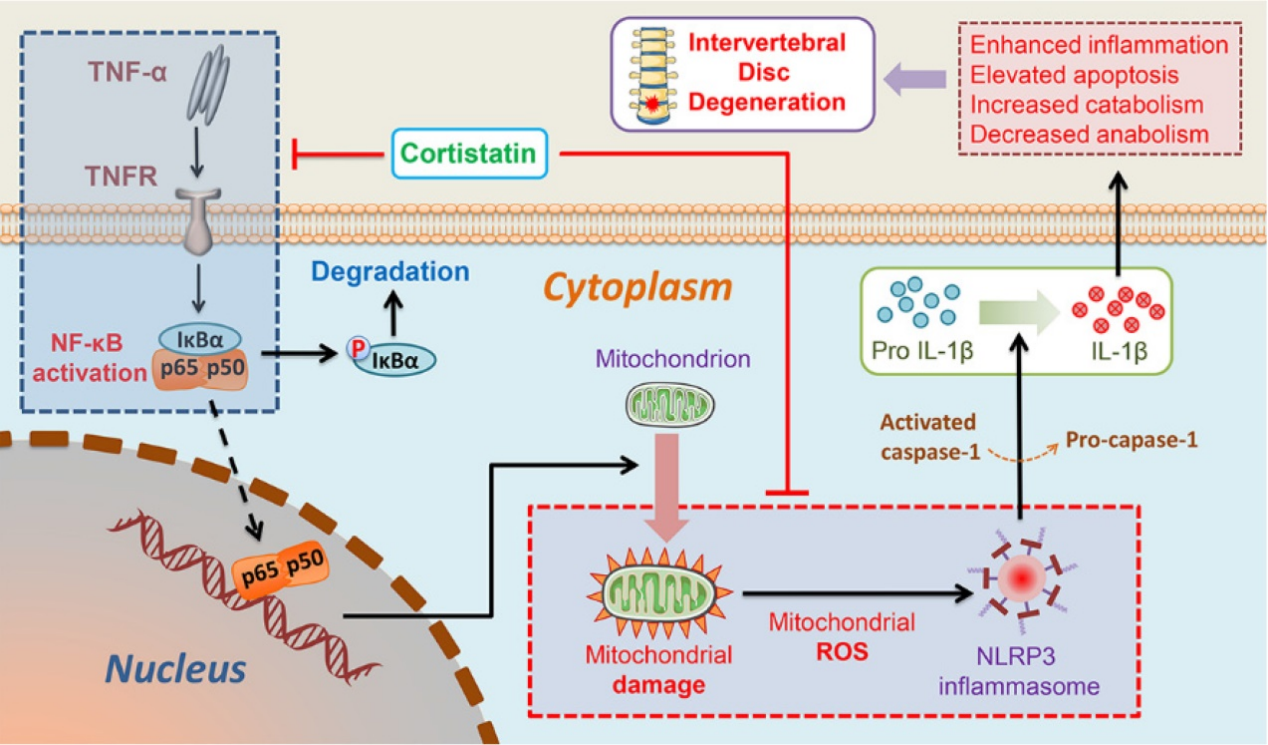
Schematic depicting a proposed model for the function of CST in intervertebral disc degeneration.

In this study, we found that the NP cells of CST-knockout mice displayed accelerated ageing, but exogenous CST effectively alleviated the cytokine-induced IVD degeneration process. In addition, the role of CST in NP cells might be associated with the regulation of mitochondrial dysfunction and activation of the NLRP3 inflammasome (**Figure 8**). Moreover, CST may interact with the TNF-α/NF-κB signalling pathway in NP cells in the ageing process (**Figure 8**). Taken together, these three findings reveal the protective role and mechanism of CST in the IVD during the ageing process from different perspectives, which might shed light on therapeutic strategies for IVD degenerative diseases.

## Supplementary Material

Supplementary figures and tables.

## Abbreviations

CST: cortistatin
IVD: intervertebral disc
NP: nucleus pulposus
WT: wild-type
CTL: control
TNF-α: tumor necrosis factor-α
ROS: reactive oxygen species
NLRP3: NLR family pyrin domain containing 3
NF-κB: nuclear factor kappa B
ECM: extracellular matrix
IL-1β: interleukin 1beta
MMP-13: matrix metalloproteinase-13
ADAMTS-5: a disintegrin and metalloproteinase with thrombospondin motif-5
iNOS: inducible nitric oxide synthase
COX-2: cyclooxygenase-2
Col 2: collagen type 2
Bax: Bcl-2-Associated X
Bcl-2: B cell lymphoma 2
TEM: transmission electron microscopy
OCR: oxygen consumption rate
ECAR: extracellular acidification rate
Drp1: dynamin-related peptide1
OPA1: optic atrophy 1
Mfn1/2: mitofusin1/2
DCFDA: 2',7'-dichlorofluorescein diacetate
AMPK: AMP-activated protein kinase
pAMPK: phosphorylated AMPK
PGC1α: peroxisome proliferator-activated receptor-γ coactivator 1α
