# Supplementary material for: Cortistatin protects against intervertebral disc degeneration through targeting mitochondrial ROS-dependent NLRP3 inflammasome activation
Source: Theranostics. 2020 May 27;10(15):7015–33. doi: 10.7150/thno.45359 (PMC7295059; doi:10.7150/thno.45359)
Supplement: Supplementary file 1 — Supplementary figures and tables. [file thnov10p7015s1.pdf]

Cortistatin protects against intervertebral disc degeneration through targeting mitochondrial ROS-dependent NLRP3 inflammasome activation

Yunpeng Zhao[1,*], Cheng Qiu[1,2,*], Wenhan Wang[1,2], Jiangfan Peng[2], Xiang Cheng[2], Yangtao Shangguan[2], Mingyang Xu[2], Jiayi Li[2], Ruize Qu[1,2], Xiaomin Chen[1,2], Suyi Jia[2], Dan Luo[3], Long Liu[4], Peng Li[4], Fengjin Guo[5], Krasimir Vasilev[6,7], Liang Liu[8], John Hayball[8,9], Shuli Dong[10], Xin Pan[1], Yuhua Li[1], Linlin Guo[2], Lei Cheng[1,¶], Weiwei Li[4,¶]

1. Department of Orthopaedic Surgery, Qilu Hospital, Cheeloo College of Medicine, Shandong University, Jinan, Shandong, 250012, P. R. China.
2. Cheeloo College of Medicine, Shandong University, Jinan, Shandong, 250012, P. R. China.
3. College of Stomatology, Qingdao University, Qingdao, Shandong, 266071, P. R. China.
4. Department of Pathology, Qilu Hospital, Cheeloo College of Medicine, Shandong University, Jinan, Shandong, 250012, P. R. China.
5. Department of Cell Biology and Genetics, Core Facility of Development Biology, Chongqing Medical University, Chongqing, 400016, P. R. China.
6. Future Industries Institute, University of South Australia, Mawson Lakes Campus, Mawson Lakes 5095, Australia.
7. School of Engineering, University of South Australia, Mawson Lakes Campus, Mawson Lakes 5095, Australia.
8. Experimental Therapeutics Laboratory, University of South Australia Cancer Research Institute, Adelaide SA 5000, Australia.
9. Robinson Research Institute and Adelaide Medical School, University of Adelaide, Adelaide SA 5005, Australia.
10. Key Laboratory of Colloid and Interface Chemistry (Shandong University), Ministry of Education, Jinan, 250100, P. R. China.
* These authors contributed equally to this work.

Running title: Role of cortistatin in IVD degeneration

¶ To whom correspondence should be addressed: Lei Cheng, Department of Orthopaedic Surgery, Qilu Hospital, Cheeloo College of Medicine, Shandong University, 107 Wenhuaxi Road, Jinan, 250012, P. R. China. Tel: +86-531-82166551; Fax: +86-531-88382044; Email: chenglei@email.sdu.edu.cn. Weiwei Li, Department of Pathology, Qilu Hospital, Cheeloo College of Medicine, Shandong University, 107 Wenhuaxi Road, Jinan, 250012, P. R. China. Tel: +86-531-82166551; Fax: +86-531-88382044; Email: lwwzyp@email.sdu.edu.cn.

This file includes:

Figure S1-S9

Table S1-S5

Figure S1

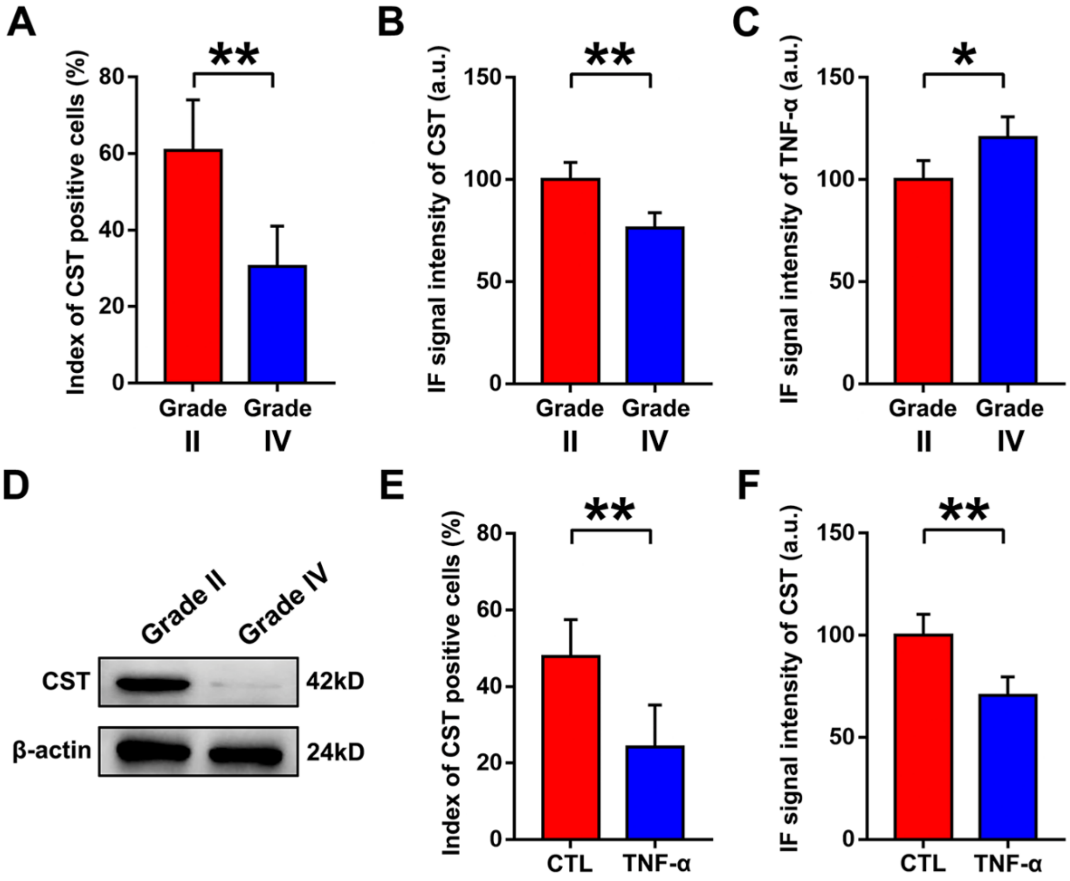

Figure S1. (A) The index of CST positive cells percentage based on immunohistochemistry of Figure 1C. **(B-C)** IF signal intensity of CST and TNF-α based on immunofluorescence of Figure 1D. **(D)** Expression of CST in NP tissues of Grade II (n=10) and Grade IV (n=7) group, as assayed through Western blot. **(E)** The index of CST positive cells percentage based on immunohistochemistry of Figure 1H (n=5). **(F)** IF signal intensity of CST and TNF-α based on immunofluorescence of Figure 1K (n=5). *p<0.05 and **p<0.01 vs. Control group. Data are presented as mean ± SD.

Figure S2

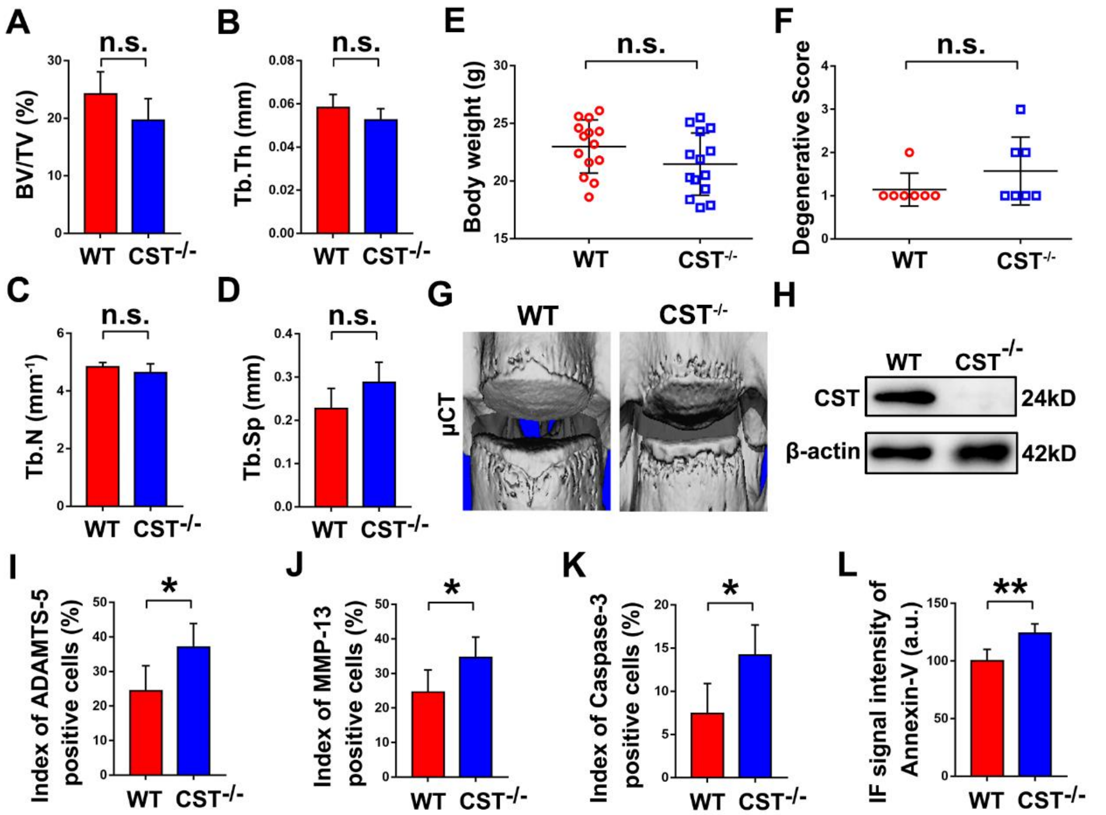

Figure S2. (A-D) The analysis of BV/TV, Tb.Th, Tb.N and Tb.Sp in vertebral body of 2-month old WT and CST$^{-/-}$ mice based on micro-CT (n=5). **(E)** Data of body weight from 2-month WT and CST$^{-/-}$ mice (n=14). **(F)** Degenerative score in IVD tissue from 2-month WT and CST$^{-/-}$ mice based on Safranin O staining (n=7). **(G)** Representative μCT images of intervertebral space region of 6-month old WT and CST$^{-/-}$ mice. Abnormal formation of osteophyte and reduction of intervertebral space could be observed in CST$^{-/-}$ mice (n=5). **(H)** No expression of CST was detected in NP tissue of CST$^{-/-}$ mice compared with WT littermates (n=3), as assayed by Western blot. **(I-J)** The percentage of ADAMTS-5 and MMP-13 positive cells based on immunohistochemistry of Figure 2F (n=5). **(K)** The percentage of caspase-3 positive cells based on immunohistochemistry of Figure 2K (n=5). **(L)** IF signal intensity of Annexin-V based on immunofluorescence of Figure 2N (n=5). n.s., not significant. *p<0.05 and **p<0.01 vs. Control group. Data are presented as mean ± SD.

Figure S3

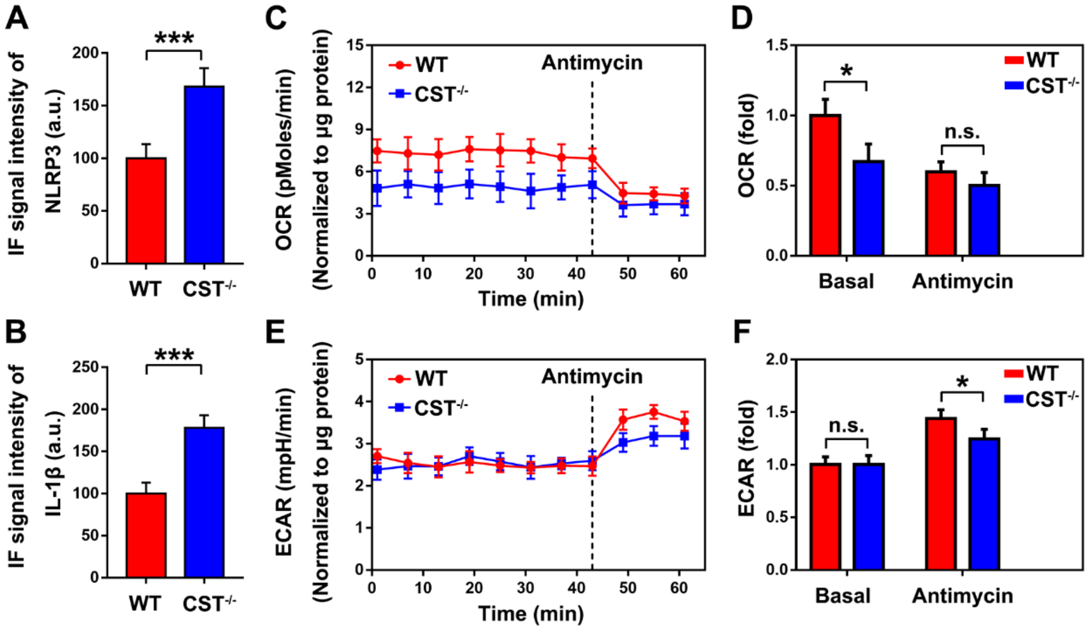

Figure S3. (A-B) IF signal intensity of NLRP3 and IL-1β based on immunofluorescence of Figure 3K (n=5). **(C-D)** Oxygen consumption rate (OCR) of NP cells reveals that CST deficiency results in the reduction of basal respiration (n=3). **(E-F)** Extracellular acidification rate (ECAR) of NP cells indicates CST deficiency results in the decreased glycolysis capacity (n=3). n.s., not significant. *p<0.05 and ***p<0.001 vs. Control group. Data are presented as mean ± SD.

Figure S4

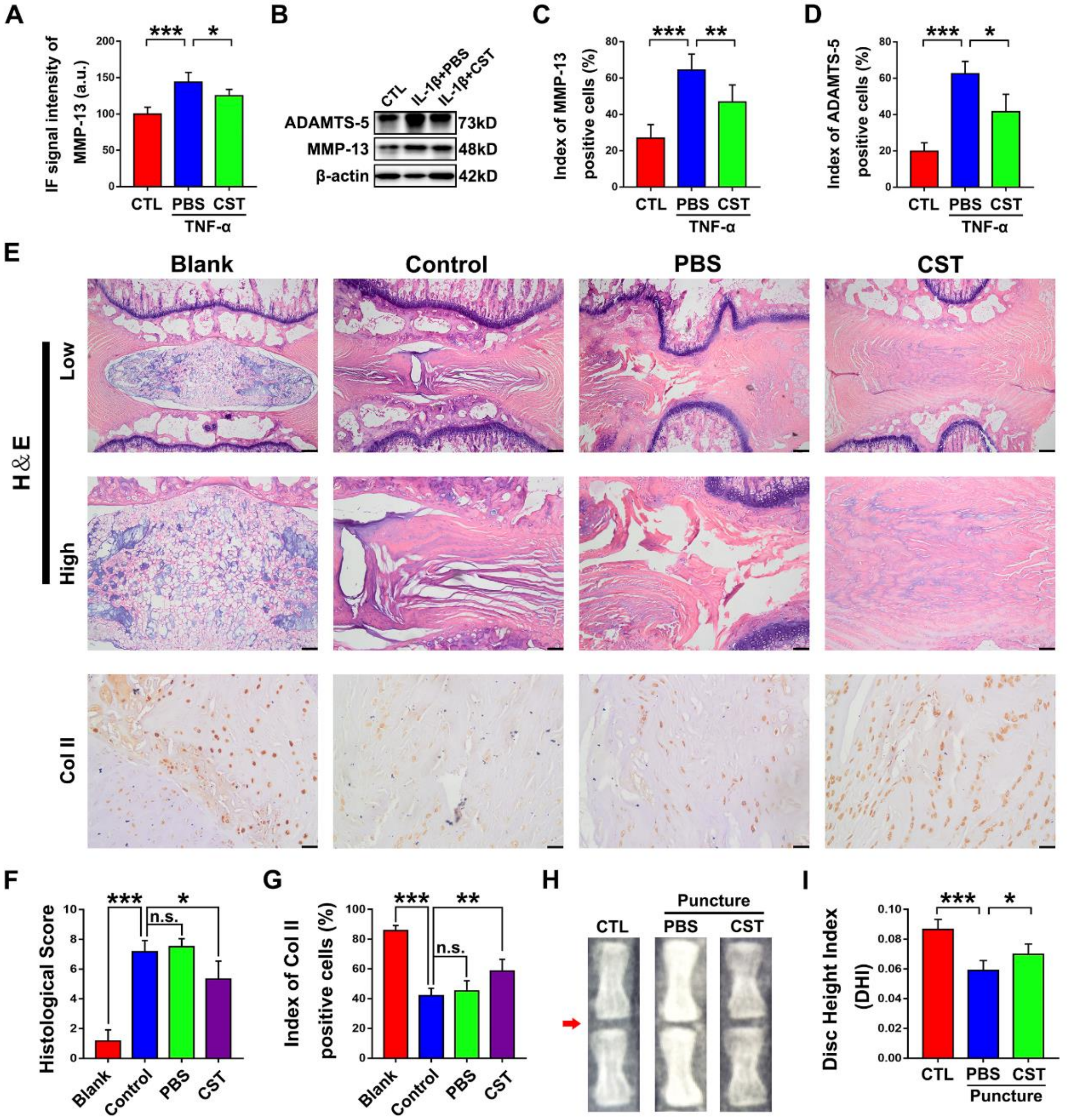

Figure S4. (A) IF signal intensity of MMP-13 based on immunofluorescence of Figure 4C (n=5). **(B)** Human NP cells stimulated by 10 ng/mL IL-1β for 24h, with or without treatment of CST recombinant peptide (50 μg/mL) and then total protein of each indicated group were extracted. CST was identified to inhibit the production of ADAMTS-5 and MMP-13 induced by IL-1β (n=5), as assayed by Western blot. **(C-D)** The index of MMP-13 and ADAMTS-5 positive cells percentage based on immunohistochemistry of Figure 4K (n=5). **(E)** Representative hematoxylin and eosin (H&E) staining and immunohistochemistry of rat IVDs (n=6). Scale bar, 150 μm (up panel), 100 μm (third and bottom panel). **(F)** Histological score of rat IVDs in each

indicated group (n=6). **(G)** Index of Col II positive cells based on immunohistochemistry of Figure S4E (n=6). **(H-I)** Representative X-ray image of each group and associated disc height index (n=6). n.s., not significant. *$p<0.05$, **$p<0.01$ and ***$p<0.001$ vs. Control group. Data are presented as mean ± SD.

Figure S5

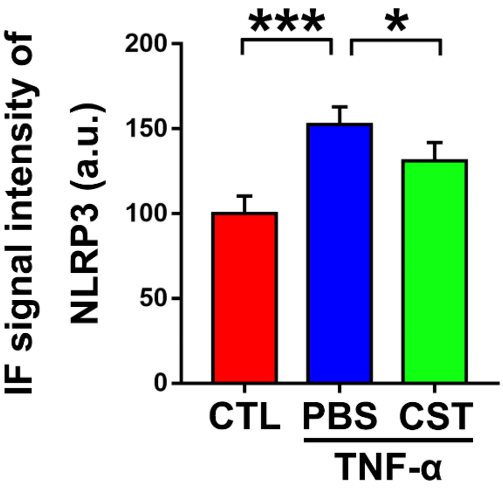

Figure S5. IF signal intensity of NLRP3 based on immunofluorescence of Figure 5G (n=5). *p<0.05 and ***p<0.001 vs. Control group. Data are presented as mean ± SD.

Figure S6

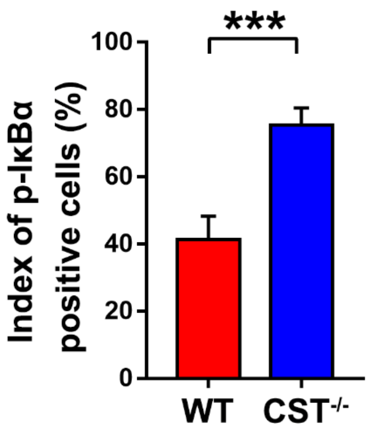

Figure S6. The index of p-IκBα positive cells percentage based on immunohistochemistry of Figure 6B (n=5). ***p<0.001 vs. Control group. Data are presented as mean ± SD.

Figure S7

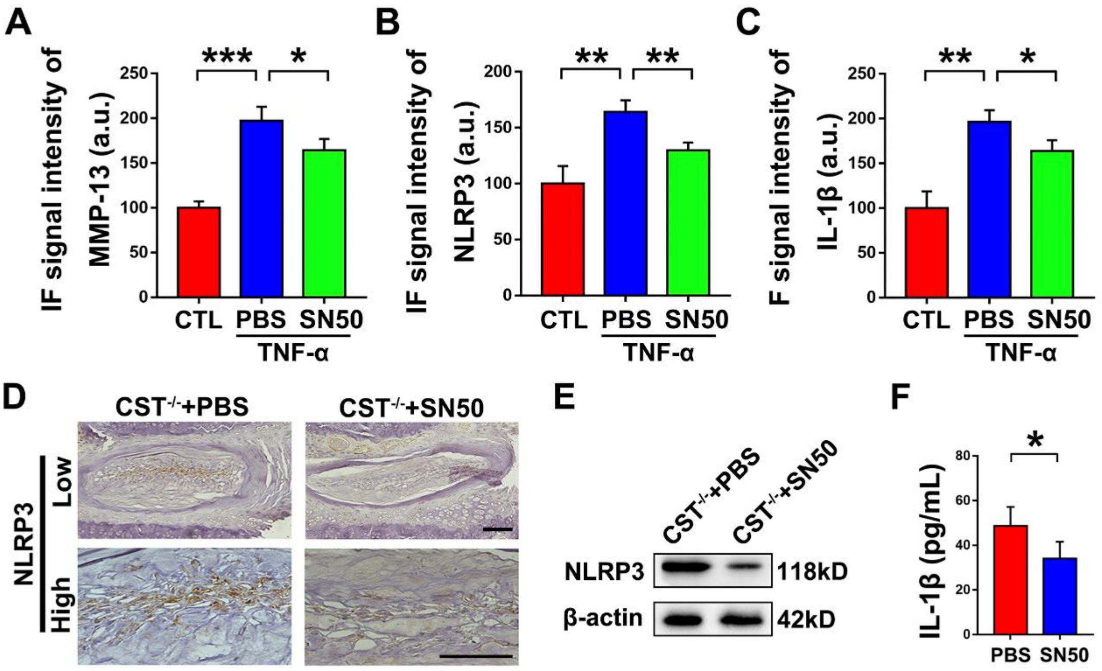

Figure S7. (A) IF signal intensity of MMP-13 based on immunofluorescence of Figure 7C (n=5). **(B-C)** IF signal intensity of NLRP3 and IL-1β based on immunofluorescence of Figure 7I (n=5). **(D)** Representative immunohistochemistry image in PBS or SN50 treated 6-month old CST$^{-/-}$ IVD tissues (n=5). Scale bar, 150 μm. **(E)** Western blot analysis of PBS and SN50 treated groups (n=5). **(F)** The expression of IL-1β in culture media of PBS and SN50 treated groups (n=5). *$p<0.05$, **$p<0.01$ and ***$p<0.001$ vs. Control group. Data are presented as mean ± SD.

Figure S8

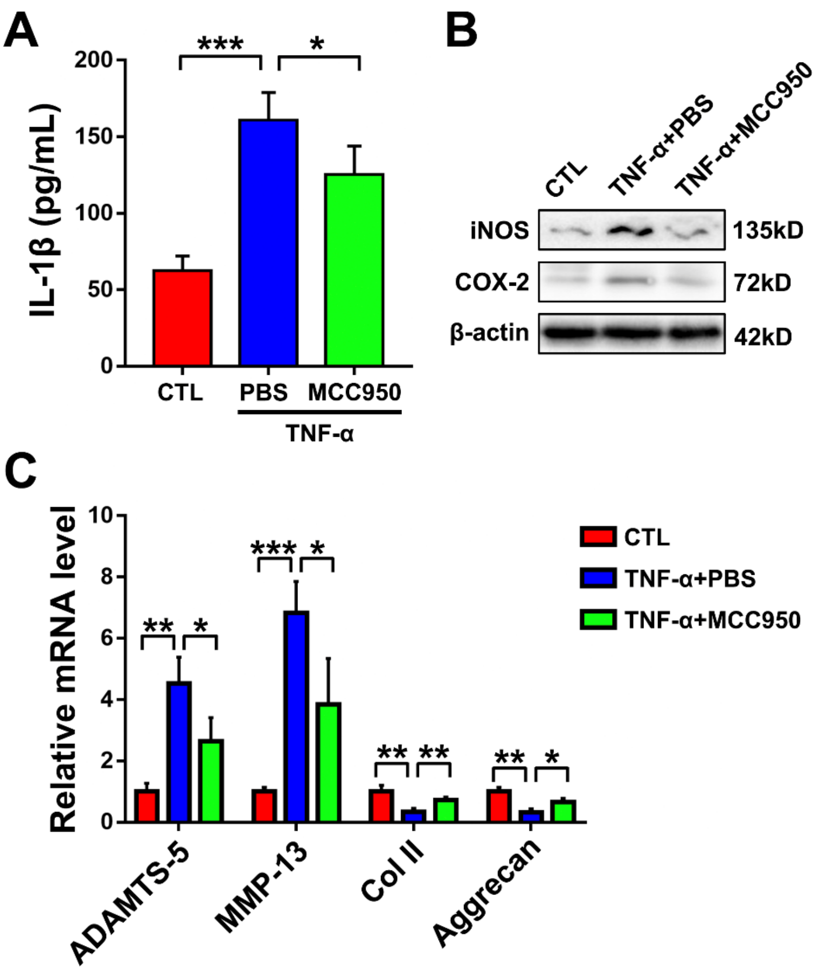

Figure S8. MCC950 attenuates impaired homeostasis of NP cells in deficiency of CST. (A) MCC950 diminished the expression of IL-1β in culture media of 2-month old murine CST$^{-/-}$ NP cells, as detected by ELISA (n=5). **(B)** Murine CST$^{-/-}$ NP cells stimulated by 10 ng/mL TNF-α for 24h, with or without treatment of 1 μM MCC950 and then total protein of each indicated group were extracted. MCC950 inhibits TNF-α-mediated expression of inflammatory mediators (iNOS and COX-2), as assayed by Western blot (n=5). **(C)** Relative mRNA expression of MMP-13, ADAMTS-5, Col 2 and Aggrecan in murine NP cells from CST$^{-/-}$ mice, as measured by Real time PCR (n=5). *p<0.05, **p<0.01 and ***p<0.001 vs. Control group. Data are presented as mean ± SD.

Figure S9

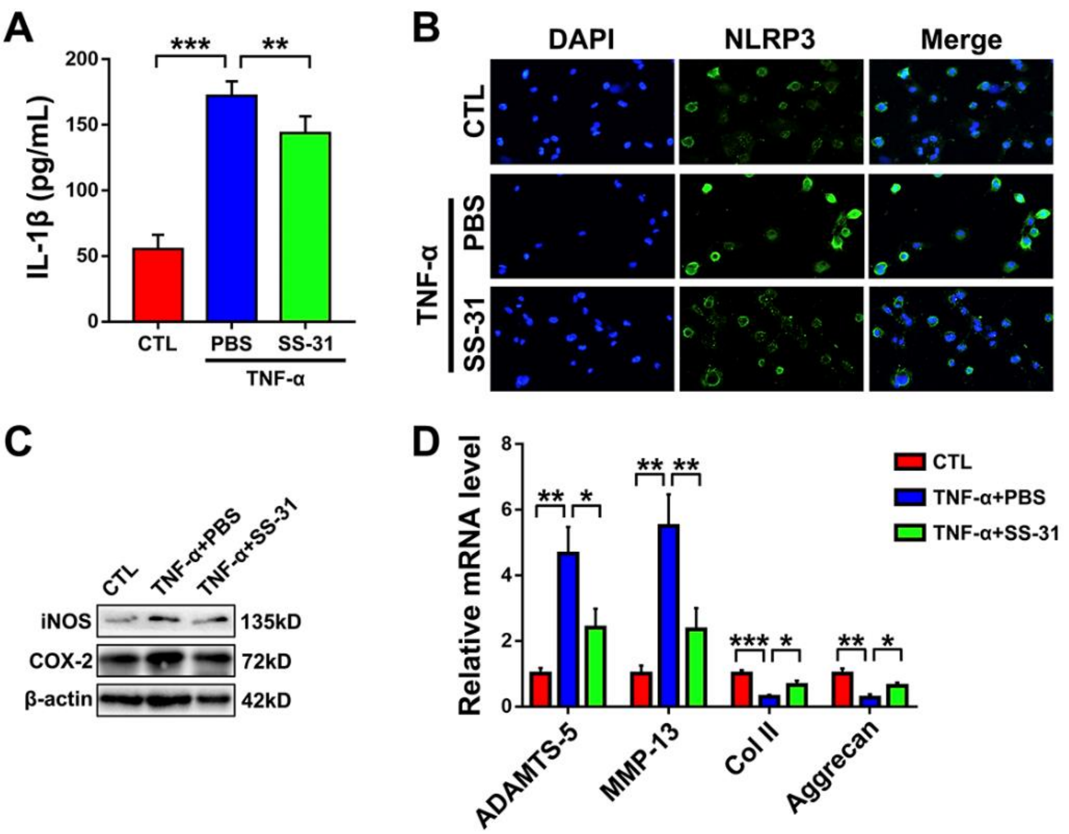

Figure S9. SS-31 alleviated NLRP3 inflammasome activation in CST$^{-/-}$ NP cells. **(A)** SS-31 markedly downregulated expression of IL-1β in culture media of 2-month old murine CST$^{-/-}$ NP cells, as detected by ELISA (n=5). **(B)** Murine CST$^{-/-}$ NP cells stimulated by 10 ng/mL TNF-α for 24h, with or without treatment of 5 μM SS-31 and then total protein of each indicated group were extracted. SS-31 attenuates the TNF-α-mediated activation of NLRP3 (n=5). **(C)** SS-31 inhibits TNF-α-mediated the expression of inflammatory mediators (iNOS and COX-2), as assayed by Western blot (n=5). **(D)** Relative mRNA expression of MMP-13, ADAMTS-5, Col 2 and Aggrecan in murine NP cells from CST$^{-/-}$ mice, as measured by Real time PCR (n=5). *p<0.05, **p<0.01 and ***p<0.001 vs. Control group. Data are presented as mean ± SD.

Table S1: Summary of clinical and demographic features of patients.

Subject Number	Gender	Age	Level	Pfirrmann grading
**Grade II group**				
1	F	30	L3/L4	II
2	M	29	L3/L4	II
3	F	28	L4/L5	II
4	M	29	L3/L4	II
5	M	24	L4/L5	II
6	F	32	L4/L5	II
7	M	24	L4/L5	II
8	F	26	L2/L3	II
9	F	41	L5/S1	II
10	M	27	L5/S1	II
**Grade IV group**				
11	M	42	L4/L5	IV
12	F	60	L5/S1	IV
13	F	51	L4/L5	IV
14	M	52	L4/L5	IV
15	F	53	L4/L5	IV
16	F	74	L5/S1	IV
17	M	65	L5/S1	IV

Table S2: Sequences of mice primers used for quantitative real-time PCR.

	Gene	Primer sequence (5'-3')
Mouse	*Cort*	Forward: GGAGCGGCCTTCTGACTTTCC<br>Reverse: GCCTTTCCTGGCTCTTGGACA
	Mmp13	Forward: ACTTTGTTGCCAATTCCAGG<br>Reverse: TTTGAGAACACGGGGAAGAC
	Adamts5	Forward: GCATTGACGCATCCAAACCC<br>Reverse: CGTGGTAGGTCCAGCAAACAGTTAC
	Nos2	Forward: ACAGGAGGGGTTAAAGCTGC<br>Reverse: TTGTCTCCAAGGGACCAGG
	Cox2	Forward: AATGCTGACTATGGCTACAAAA<br>Reverse: AAAACTGATGCGTGAAGTGCTG
	Col2a1	Forward: ACTAGTCATCCAGCAAACAGCCAGG<br>Reverse: TTGGCTTTGGGAAGAGAC
	Acan	Forward: AATGCTGGTACTCCAAACCC<br>Reverse: CTGGATCGTTATCCAGCAAACAGC
	Casp3	Forward: AGGAGGGACGAACACGTCT<br>Reverse: CAAAGAAGGTTGCCCCAATCT
	Bax	Forward: CTGAGCTGACCTTGGAGC<br>Reverse: GACTCCAGCCACAAAGATG
	Bcl2	Forward: TGTGGTCCATCTGACCCTCC<br>Reverse: ACATCTCCCTGTTGACGCTCT
	Nlrp3	Forward: GAGTTCTTCGCTGCTATGT<br>Reverse: ACCTTCACGTCTCGGTTC
	Nfkb2	Forward: TACAAGCTGGCTGGTGGGGA<br>Reverse: GTCGCGGGTCTCAGGACCTT
	Gapdh	Forward: CTTCACCACCATGGAGAAGGC<br>Reverse: GACGGACACATTGGGGGTAG

Table S3: Sequences of human primers used for quantitative real-time PCR.

	Gene	Primer sequence (5'-3')
**Human**	*CORT*	Forward: CGTGTCTTGAGTAATTTGGA
		Reverse: ATGAACATCAGAAGAAAAGC
	ADAMTS5	Forward: GCAGTATGACAAGTGCGGAGT
		Reverse: CAGGGCTAAATAGGCAGTGAA
	MMP13	Forward: ACTTTGTTGCCAATTCCAGG
		Reverse: TTTGAGAACACGGGGAAGAC
	COL2A1	Forward: TGAGGGCGCGGTAGAGACCC
		Reverse: TGCACACAGCTGCCAGCCTC
	ACAN	Forward: AATGCTGGTACTCCAAACCC
		Reverse: CTGGATCGTTATCCAGCAAACAGC
	CASP3	Forward: GAGCACTGGAATGTCATCTCGCTCTG
		Reverse: AGACCGAGATGTCATTCCAGTGCTT
	BAX	Forward: CCCGAGAGGTCTTTTTCCGAG
		Reverse: CCAGCCCATGATGGTTCTGAT
	BCL2	Forward: ACTTCGCCGAGATGTCC
		Reverse: ATGACCCCACCGAACTC
	GAPDH	Forward: AGAAGGCTGGGGCTCATTTG
		Reverse: AGGGGCCATCCACAGTCTTC

Table S4: Antibodies used for Western Blot.

Name	Source	Catalog number	Dilution
Anti-cortistatin	Santa Cruz Biotechnology	sc-393108	1:1000
Anti-MMP-13	Santa Cruz Biotechnology	sc-515284	1:1000
Anti-ADAMTS-5	Abcam	ab41037	1:500
Anti-iNOS	Proteintech	18985-1-AP	1:500
Anti-COX-2	Boster	BA0738	1:1000
Anti-Col 2	Santa Cruz Biotechnology	sc-52658	1:1000
Anti-aggrecan	Proteintech	13880-1-AP	1:1000
Anti-caspase-3	Proteintech	19677-1-AP	1:2000
Anti-Bax	Boster	BM3964	1:400
Anti-Bcl-2	Abcam	ab196495	1:1000
Anti-OPA1	Boster	PB0773	1:2000
Anti-Drp1	Proteintech	12957-1-AP	1:3000
Anti-Mfn1	Proteintech	13798-1-AP	1:1000
Anti-Mfn2	Proteintech	12186-1-AP	1:3000
Anti-pAMPK	Affinity Biosciences	AF3422	1:2000
Anti-AMPK	Affinity Biosciences	AF6423	1:2000
Anti-PGC1α	Proteintech	66369-1-Ig	1:3000
Anti-NLRP3	Abcam	ab214185	1:1000
Anti-NF-κB p65	Proteintech	10745-1-AP	1:3000
Anti-Tubulin	Proteintech	66240-1-Ig	1:15000
Anti-Lamin A	Santa Cruz Biotechnology	sc-71481	1:1000
Anti-IκBα	Abcam	ab32518	1:3000
Anti-pIκBα	Santa Cruz Biotechnology	sc-8404	1:1000
Anti-β-actin	Abcam	60008-1-Ig	1:10000

Table S5: Antibodies used for Immunohistochemistry.

Name	Source	Catalog number	Dilution
Anti-cortistatin	Santa Cruz Biotechnology	sc-393108	1:200
Anti-MMP-13	Santa Cruz Biotechnology	SC-515284	1:200
Anti-ADAMTS-5	Abcam	ab41037	1:200
Anti-caspase-3	Proteintech	19677-1-AP	1:200
Anti-NLRP3	Abcam	ab214185	1:100
Anti-p-IκBα	Santa Cruz Biotechnology	sc-8084	1:100
